# Synthesis of broad-specificity activity-based probes for *exo*-β-mannosidases†

**DOI:** 10.1039/d1ob02287c

**Published:** 2021-12-23

**Authors:** Nicholas G. S. McGregor, Chi-Lin Kuo, Thomas J. M. Beenakker, Chun-Sing Wong, Wendy A. Offen, Zachary Armstrong, Bogdan I. Florea, Jeroen D. C. Codée, Herman S. Overkleeft, Johannes M. F. G. Aerts, Gideon J. Davies

**Affiliations:** York Structural Biology Laboratory, Department of Chemistry, The University of York York YO10 5DD UK gideon.davies@york.ac.uk; Department of Bio-Organic Chemistry, Leiden Institute of Chemistry, Leiden University P. O. Box 9502 2300 RA Leiden The Netherlands; Department of Medical Biochemistry, Leiden Institute of Chemistry, Leiden University P. O. Box 9502 2300 RA Leiden The Netherlands

## Abstract

*Exo*-β-mannosidases are a broad class of stereochemically retaining hydrolases that are essential for the breakdown of complex carbohydrate substrates found in all kingdoms of life. Yet the detection of *exo*-β-mannosidases in complex biological samples remains challenging, necessitating the development of new methodologies. Cyclophellitol and its analogues selectively label the catalytic nucleophiles of retaining glycoside hydrolases, making them valuable tool compounds. Furthermore, cyclophellitol can be readily redesigned to enable the incorporation of a detection tag, generating activity-based probes (ABPs) that can be used to detect and identify specific glycosidases in complex biological samples. Towards the development of ABPs for *exo*-β-mannosidases, we present a concise synthesis of β-*manno*-configured cyclophellitol, cyclophellitol aziridine, and *N*-alkyl cyclophellitol aziridines. We show that these probes covalently label *exo*-β-mannosidases from GH families 2, 5, and 164. Structural studies of the resulting complexes support a canonical mechanism-based mode of action in which the active site nucleophile attacks the pseudoanomeric centre to form a stable ester linkage, mimicking the glycosyl enzyme intermediate. Furthermore, we demonstrate activity-based protein profiling using an *N*-alkyl aziridine derivative by specifically labelling MANBA in mouse kidney tissue. Together, these results show that synthetic *manno*-configured cyclophellitol analogues hold promise for detecting *exo*-β-mannosidases in biological and biomedical research.

## Abstract 2

New cyclophellitol-derived probes covalently label β-mannosidases, enabling the detection of diverse mannosidases *via* activity-based protein profiling.

## Graphical abstract

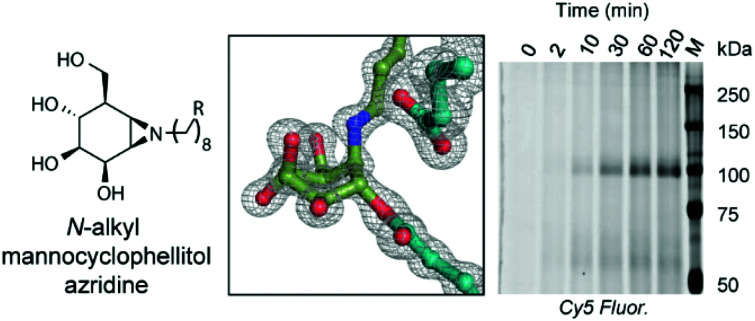

## Introduction

*Exo*-β-mannosidases, hydrolysing non-reducing terminal β-d-mannopyranosidic linkages, are widely distributed carbohydrate-degrading enzymes.^1^ In the human gut, *exo*-β-mannosidases of families GH2, GH5, and GH164 are essential for the fermentation of β-mannans, such as carob or guar endosperm (*Ceratonia siliqua*) galactomannan, common food additives.^2–5^ In mammals, the lysosomal glycoside hydrolase family 2 (GH2 ^6^) retaining *exo*-β-mannosidase, MANBA (EC 3.2.1.25), is responsible for the cleavage of the core Man-β-1,4-GlcNAc linkage during lysosomal turnover of *N*-glycoproteins.^7^ Deficiency of this enzyme activity, caused by mutations in MANBA,^8,9^ results in β-mannosidosis, a rare lysosomal storage disorder that can be diagnosed on the basis of elevated Man-β-1,4-GlcNAc in urine or diminished hydrolysis of 4-methylumbelliferyl (4-MU) β-d-mannopyranoside in plasma.^10–12^ More recently, MANBA deficiency has been identified as a key risk factor for chronic kidney disease, which affects roughly 1 in 10 adults.^13–15^

**Fig. 1:**
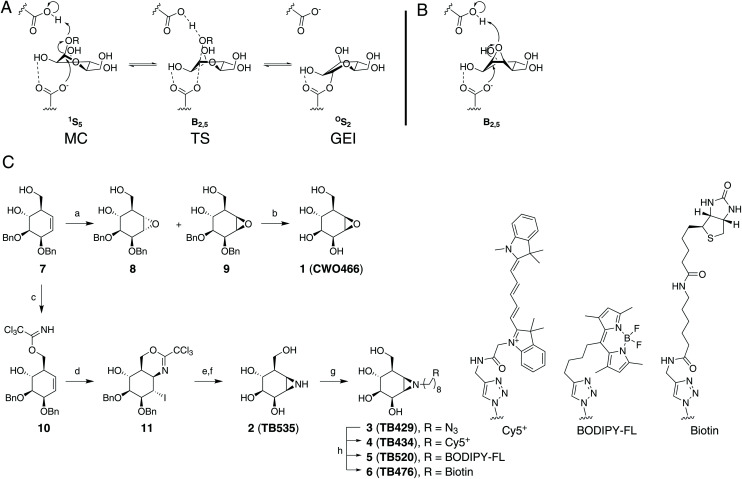
(A) Conformational reaction itinerary employed by GH2 β-mannosidases in the glycosylation half-reaction. MC: Michaelis complex, TS: transition state; GEI: glycosyl-enzyme intermediate (B) expected reactive binding conformation of compound 1. (C) Synthesis of the target molecules used in this study. (a) *m*CPBA, DCE, reflux (8: 38%, 9: 26%); (b) Pd/C, H_2_, 1,4-dioxane/*t*BuOH (9 : 1), r.t., 40%; (c) Cl_3_CCN, DBU, DCM, 0 °C to r.t., 30 min, 73%; (d) I_2_, NaHCO_3_, THF/H_2_O (4 : 1), 60 °C, 80%; (e) i. 1,4-dioxane/H_2_O/AcOH (1 : 1 : 8), r.t., 16 h, 75%; ii. NaHCO_3_, MeOH, r.t., 16 h, 58%; (f) Li, NH_3_ (liq), −60 °C, 30 min, quant.; (g) 1-azido-8-iodooctane,^29^ K_2_CO_3_, DMF, 80 °C, overnight, 64%; (h) Cy5 alkyne, BODIPY-FL-alkyne,^30^ or biotin alkyne,^31^ CuSO_4_, sodium ascorbate, H_2_O/DMF, r.t., overnight (4: 17%, 5: 36%, 6: 32%).

All of these *exo*-β-mannosidases process their substrate using a stereochemically retaining double displacement mechanism that is characterised by the formation of a covalent glycosyl enzyme intermediate (GEI, Fig. 1A). This two-step mechanism, first outlined by Koshland,^16^ can be exploited in the development of activity-based probes (ABPs). The natural product cyclophellitol, with endocyclic epoxide functionality in place of the acetal group found in a typical glycoside, is a potent irreversible inhibitor of retaining β-glucosidases.^17,18^ It has been demonstrated that altering the characteristic cyclitol ring to emulate differently configured monosaccharides yields irreversible inhibitors with selectivity towards various retaining *exo*-glycosidases.^19–24^ Moreover, it has been shown that substituting the epoxide in cyclophellitol and its configurational isosteres for an *N*-tagged (*e.g.* fluorophore, biotin) aziridine yields effective ABPs for the discovery and visualisation of retaining glycosidases within complex biological samples.^19,25–28^

The inhibition of *exo*-β-mannosidases using cyclophellitol derivatives presents a unique challenge. GH2 *exo*-β-mannosidases process their substrate through a ^1^S_5_–B_2,5_^‡^–^O^S_2_ mechanistic conformational itinerary (Fig. 1A).^32^ Due to the strain of the three-membered ring, cyclophellitols strongly favour ^4^H_3_ or ^3^H_4_ half-chair conformations similar to mannoimidazole, a non-covalent transition state mimic.^1,33^ This matches the substrate conformation of the β-glucosidase transition-state, but presents a significant energy barrier to reaching the *exo*-β-mannosidase ^1^S_5_ pre-catalytic or B_2,5_ transition state conformations (Fig. 1B).^33,34^ Thus, cyclophellitol-derived ABPs may not be able to efficiently label *exo*-β-mannosidases. Furthermore, such a probe might not show selectivity towards *exo*-β-mannosidases over *exo*-β-glucosidases for the same reason. However, we were encouraged by recent work showing that Golgi α-mannosidase II, using a ^O^S_2_–B_2,5_^‡^–^1^S_5_ conformational itinerary, could be efficiently probed using an alkylaziridine warhead.^23^

## Results and discussion

### Synthesis of manno-configured cyclophellitols

To determine whether conformational mismatch precludes *exo*-β-mannosidase inhibition and labelling, we first established an efficient synthesis, rooted in the concise Madsen synthesis of cyclophellitol.^23,35^ As for the manno-epi-cyclophellitols, the synthesis was initiated from d-ribose instead of d-xylose to obtain the necessary stereochemistry (see Fig. 1C for synthetic approach).^23^ To generate compound 1, key cyclohexene 7 (generated from appropriately protected d-ribose in 5 steps) was epoxidized with *m*CPBA, furnishing partially protected β-d-*manno*-cyclophellitol alongside the epimeric epoxide 8. Purification by silica gel chromatography yielded pure 9, which was subsequently deprotected by palladium-catalysed hydrogenolysis in a 9 : 1 mixture of 1,4-dioxane and ^*t*^BuOH to provide β-d-*manno*-cyclophellitol 1 in 40% yield after compound crystallisation.

To prepare aziridine 2, the intramolecular iodocyclization route was chosen over the epoxide opening (with sodium azide) and closing (under reducing conditions), to take advantage of the stereochemistry of the primary hydroxyl group directing to the beta face. Starting cyclohexene 7 was thus treated with trichloroacetonitrile to furnish the trichloroacetimidate 10 in 73% yield, which was subjected to iodocyclisation to give 11 in 80% yield. Acid-mediated hydrolysis of the resulting iminal and base-induced intramolecular displacement of the iodine with the liberated primary amine gave the partially benzylated aziridine 12. This was reduced under Birch conditions to give β-d-*manno*-cyclophellitol aziridine 2 in quantitative yield.

Treatment of 2 with 1-azido-8-iodooctane^29^ and potassium carbonate in DMF yielded the *N*-alkyl aziridine 3 in 64% yield. The introduced azide handle was further reacted with Cy5^+^ alkyne, BODIPY-FL-alkyne^30^ or biotin–alkyne^31^ under copper(i)-catalysed azide–alkyne [2 + 3] cycloaddition ‘click’ conditions to afford final ABPs 4, 5, and 6, respectively, in 17 to 36% yield (see ESI† for experimental details on the synthesis and analytical details for the intermediates and final products).

### Manno-configured cyclophellitols are covalent inhibitors of *exo*-β-mannosidases

Having candidate inhibitors 1–3 and activity-based probes 4–6 we initially tested 1, 2, and 3 against a diverse collection of well-characterised bacterial *exo*-β-mannosidases, including *Bt*Man2A,^4^*Cm*Man5A,^2^ and *Bs*GH164.^5^ Where covalent inhibition was observed, it was extremely slow. Activity and intact MS measurements collected following 25 hours incubation of each enzyme with 1 mM of each inhibitor under optimal conditions showed distinct patterns of reactivity for each enzyme (ESI Fig. 1 and Table 1†). *Bs*GH164 did not react with *N*-octylazido aziridine 3, but was labelled by both epoxide 1 (76% inhibition) and aziridine 2 (30% inhibition). *Cm*Man5A showed 20% inhibition with both 1 and 2, and stronger 85% inhibition with 3, indicating that binding is enhanced by the presence of the alkyl group. No significant inhibition of *Bt*Man2A could be detected following incubation with 1 or 2, but the enzyme was 85% inhibited by 3, again indicating performance enhancement attributable to the *N*-octylazido linker. Assuming a second-order reaction model between the enzyme and inhibitor, these measurements imply extremely low *k*_inact_/*K*_I_ values for the interaction ranging from <0.004–0.034 M^−1^ s^−1^_._

**Fig. 2:**
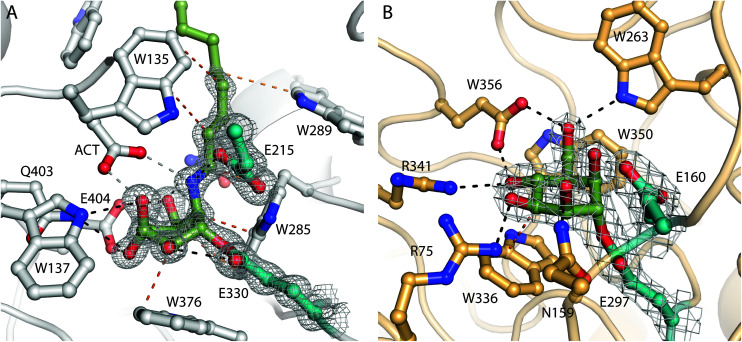
(A) Structure of *Cm*Man5A labelled with compound 3. The catalytic nucleophile and general acid/base are shown in teal, other active site residues (and the bound acetate, ACT) are shown in white, and compound 3 is shown in green. 2*F*_o_ − *F*_c_ electron density, contoured to 1.5*σ*, is shown around compound 3 and the catalytic residues. Apparent hydrogen bonding interactions are shown as black dashed lines while apparent hydrophobic close contacts are shown as orange dashed lines. (B) Structure of *Bs*GH164 following labelling with compound 1. The catalytic nucleophile, general acid/base, ligand, interactions, and electron density are shown as in panel A. Other active site residues are shown in light orange.

To investigate the structural basis of the observed binding, we prepared covalent crystallographic complexes between each enzyme and its most reactive ligand. *Cm*Man5A bound to compound 3 gave the highest resolution structure at 1.3 Å. Supporting our hypothesized mechanism of reactivity, compound 3 was covalently linked to E330, the known catalytic nucleophile, through its pseudoanomeric carbon (Fig. 2A). The ring was found in an ^O^S_2_-like conformation, providing the first direct evidence that the GEI conformation for GH5 β-mannosidases matches that of GH2 β-mannosidases^36^ and GH26 β-mannanases.^37^ The amine group from the opened aziridine is found interacting with both the general acid, E215, and an apparent buffer acetate that also interacts with the axial O3. C7 (taking the place of endocyclic oxygen) forms a CH/π interaction (3.4 Å) with W285 while the alkyl chain extends out of the catalytic pocket, forming hydrophobic packing interactions with W285, W135, and a hydrophobic groove between N288 and W289. We speculate that these interactions contribute to the observed tighter binding of compound 3 over compound 2. Indeed, the first four carbons of the alkyl linker display well-localised electron density (B factors of 16–25 Å^2^), indicating rigid binding and suggesting that a shorter linker may hinder binding into the active site pocket.

In contrast, the structure of *Bt*Man2A labelled with 3, solved at 1.85 Å resolution, showed the ligand bound to the catalytic nucleophile in a “relaxed” ^4^C_1_-like conformation (ESI Fig. 2A†). The electron density for several aromatic residues lining the entrance to the active site pocket, notably W470, W533, and Y537 were extremely diffuse, hindering modelling and suggesting a high degree of mobility in the complex. The alkyl linker extends into a channel lined with these diffuse aromatic residues, possibly giving rise to similar, albeit more dynamic, hydrophobic interactions to those observed in the *Cm*Man5A active site. Comparisons of the labelled enzyme structure to the previously determined structures of complexes with 2,4-dinitrophenyl 2-deoxy-2-fluoro-β-d-mannoside (2FMan, non-covalent/pre-catalytic) and noeuromycin,^36^ show that the ligand has migrated significantly away from the expected position of the GEI (ESI Fig. 3†). Reorientation of the catalytic nucleophile breaks the hydrogen bond with O2, allowing relaxation of the ring conformation. This makes space for a water molecule to interact with R393. Such reorientation is accompanied by the displacement of W533 and Y537. Due to the interaction of these aromatic rings with the alkyl linker, we hypothesized that hydrophobic interactions related to the presence of the octyl linker drive this active site rearrangement. To test this hypothesis, we performed an extensive soak (10 mM, 4 days) of *Bt*Man2A crystals with compound 2. Surprisingly, the resulting electron density map showed similar diffusion of electron density and rearrangement of the active site, with the ligand observed further displaced from the expected GEI in a highly unusual ^1^C_4_-like ring conformation in molecule A (ESI Fig. 2B†). In molecule B the ligand is 4*C*1-like (similar to the complex with the alkyl aziridine 3) and part unreacted aziridine (at occupancies 0.4 and 0.6 respectively). We thus conclude that there exists a more fundamental instability in the GEI structure of *Bt*Man2A, possibly explaining earlier failures to trap it.

Soaking *Bs*GH164 crystals with 1 or 2 (1 mM, 3 days, RT, pH 6.5) resulted in the formation of clear covalent adducts (Fig. 2B). Both complexes have the ligand in the same position, sharing a ^4^C_1_/E_5_ conformation as observed for the complex with 2FMan (ESI Fig. 4†).^5^ H260 shows a slight displacement in the complex with compound 2. We attribute this to Coulomb repulsion with the aziridine nitrogen, possibly explaining the weaker affinity of the aziridine with the *Bs*GH164 active site in spite of an expected higher electrophilicity. The structure presents no obvious basis for the lack of reactivity with compound 3 as the pocket is quite open around the aziridine nitrogen. In fact, an ethylene glycol monomer is observed bound where the linker could reasonably be accommodated.

**Fig. 3:**
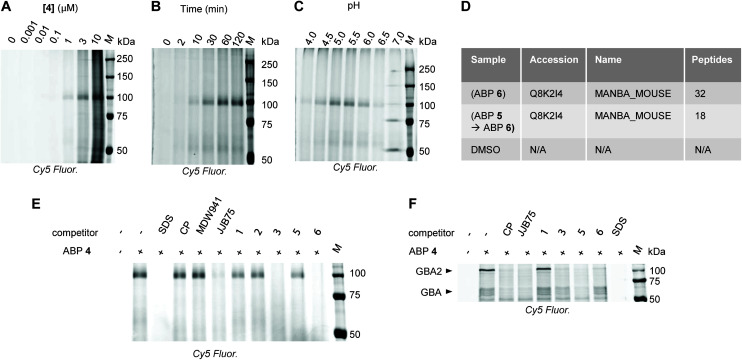
(A–C) Concentration-, time-, and pH-dependence (see ESI† for full incubation conditions) of MANBA labelling with ABP 4 in mouse kidney extracts. (D) Pulldown proteomic detection of MANBA from mouse kidney extracts. (E) cABPP of MANBA in mouse kidney extracts. SDS indicates denaturation with 2% (w/v) SDS prior to labelling, CP indicates cyclophellitol, MDW941 ^39^ and JJB75 ^26^ are established probes for GBA, *M* indicates molecular weight ladder. (F) cABPP of GBA and GBA2 in HEK293 cells. Labels are the same as in E.

With the knowledge that GH2 and GH5 *exo*-β-mannosidases can be inhibited with compound 3, we sought to assess the potential of this molecular architecture for activity-based protein profiling (ABPP). ABPs 4, 5, and 6 were prepared and applied to the detection of MANBA in mouse kidney tissue, where MANBA is known to be highly expressed,^38^ or in human plasma, where low levels of detected MANBA activity can be used to rule out a diagnosis of β-mannosidosis. ABPP, *via* in-gel fluorescence following SDS-PAGE separation, of diluted and pH-adjusted plasma from a healthy donor (measured MANBA activity = 0.07 ± 0.002 mU mL^−1^ against 4MU-β-Man at pH 5.5, 22 °C) showed no evidence for a band at ∼100 kDa, where MANBA is expected to appear (ESI Fig. 6†). The only apparent band ran at 53 kDa and reacted preferentially with **JJB376** (a β-glucosidase probe^25^). In contrast, mouse kidney extracts treated with Cy5^+^-labelled ABP 4 yielded one major band at ∼100 kDa (Fig. 3A–C), consistent with MANBA. Treatment at various concentrations with different incubation times and pH conditions demonstrated detectable labelling above background with 1 μM probe after 10 minutes at pH values between 4.5 and 6.0.

To confirm the identity of the labelled enzyme, mouse kidney protein extract was treated with DMSO control or 3 μM biotinylated ABP 6 with or without pre-incubation with 5 μM BODIPY-FL-ABP 5 for one hour. Pull-down of biotinylated proteins followed by on-bead trypsinolysis and LC-MS/MS analysis of the resulting peptides identified MANBA as the only detected glycosidase, with fewer peptides detected following pre-incubation with BODIPY-FL-ABP 5 (Fig. 3D). Confirmation of reactivity with MANBA shows that ABP 4 reacts selectively and suggests that reactivity with mouse MANBA is relatively strong. It remains to be seen whether this relatively strong reactivity of mouse MANBA with 4 and 5 can be extended to other species, though our results with human plasma do not support the hypothesis that this reactivity extends to human MANBA.

Consistent with our results with *Bt*Man2A, competitive ABPP (cABPP) in mouse kidney extracts revealed no apparent inhibition of MANBA by 1 or 2 at 50 μM following two hours of incubation at 37 °C, as did cABPP with cyclophellitol or click-labelled 6-deoxy-6-azido cyclophellitol **MDW941.**^39^ However, **JJB75**, a potent *N*-alkyl aziridine inhibitor of β-glucocerebrosidase (GBA),^26^ proved to be a competitor for the active site of MANBA at 3 μM, suggesting that, similar to *Bt*Man2A, MANBA reactivity is significantly driven by affinity for the *N*-alkyl linker.

To further test the specificity of probe 4, lysates prepared from HEK293T cells overexpressing non-lysosomal glucocerebrosidase (GBA2) were stained with Cy5-ABP (4) (Fig. 3F), revealing apparent reactivity (qualitatively similar to previously reported labelling with **JJB75** ^26^) with both GBA and GBA2, giving detectable labelling with 1 μM compound 4. Competitive ABPP with 1 μM of either cyclophellitol or **JJB75** supported the assignment of these bands as GBA and GBA2 since **JJB75** and cyclophellitol are known to be efficient inhibitors of GBA and GBA2. Together, these results indicate that the selectivity of an assay for MANBA in mammalian tissue using probe 4 can be enhanced through pre-incubation of the sample with cyclophellitol to eliminate interference from GBA and GBA2 without reducing signal from MANBA.

## Conclusions

We have demonstrated that, as has been previously shown for other retaining glycosidases, *exo*-β-mannosidases can be inhibited covalently and irreversibly with appropriately configured cyclophellitol analogues, particularly those designed with *N*-alkyl aziridine warheads. Our structural work shows that the epoxide and aziridine inhibitors bind *Bs*GH164 in the same conformation found as 2FMan and that the *N*-alkyl aziridine binds to *Cm*Man5A in the expected ^O^S_2_-like conformation of the GH5 *exo*-β-mannosidase GEI. While exhibiting low potencies, the inhibitors and ABPs presented here react irreversibly with the catalytic nucleophile, displaying sufficient stability and specificity to enable near-quantitative labelling of the catalytic nucleophile. In spite of their limited reactivity, tagged *N*-alkyl aziridine derivatives were moreover successfully applied to ABPP in both chemical proteomics and in-gel fluorescence formats, as shown by detection, enrichment, and identification of MANBA from mouse kidney extracts.

## Experimental details

### Materials and instrumentation

All chemicals were purchased from Sigma Aldrich unless otherwise specified. β-d-Mannopyranose-configured cyclophellitol was synthesized as described in the supplemental synthetic methods and compound characterization section.

### Recombinant enzyme production

*Bs*GH164 was expressed and purified as in Armstrong and Davies.^5^ Briefly, a 1 ml starter culture of pET28-His6-*Bs*GH164 in BL21(DE3) gold cells was used to inoculate NZYTech autoinduction media (NZYTech) containing 50 μg ml^−1^ of kanamycin. Expression cultures were grown at 37 °C with shaking at 250 rpm for 6 h, the temperature was then decreased to 20 °C and cultures were incubated for an additional 22 h. Expression cultures were harvested by centrifugation (5000*g*, 30 min, 4 °C) and cell pellets were stored at −70 °C until purification. Cell pellets were resuspended in 120 ml of 50 mM HEPES, 30 mM imidazole, 200 mM NaCl, pH 7.4 with additional protease inhibitor (4-(2-aminoethyl)benzenesulfonyl fluoride, 0.1 mM), lysozyme, and DNase. Resuspended cells were then lysed by passage through a cell-disruptor homogenizer at 25 kpsi. Cell lysate was clarified by centrifugation (18 000*g*, 30 min, 4 °C) then loaded directly onto a 5 ml His-tag Excel column (GE Healthcare). Bound protein was eluted with a linear gradient of 0–100% 50 mM HEPES, pH 7.4, 1 M imidazole, 200 mM NaCl over 20 column volumes, concentrated with a 30 kDa cut-off Amicon centrifugal filter unit, and further purified by gel filtration (HiLoad 16/600 Superdex 200 pg; GE Healthcare) in 50 mM HEPES, 200 mM NaCl, pH 7.4. The purity of eluted protein was analyzed by SDS-PAGE and protein-bearing fractions were pooled and concentrated with a 30 kDa cutoff Amicon centrifugal filter unit. Concentrated protein was diluted with 20 mM HEPES, pH 7.4, concentrated again, then diluted to 30 mg ml^−1^ with the same buffer and flash frozen with liquid nitrogen until use. Protein concentrations were determined spectrophotometrically using a calculated *ε*_280_ of 128 480 M^−1^ cm^−1^.

The gene for *Cm*Man5A^2^ was codon optimized for *E. coli*, synthesized and cloned into pET24a between the NdeI and XhoI restriction sites by Genscript (Netherlands) to add a C-terminal 6xHis tag, matching the previously crystallized form. The plasmid was transformed into BL21(DE3) gold cells and expressed in ZYM-5052 autoinduction medium^40^ with 50 μg ml^−1^ of kanamycin, 2 mM MgSO_4_ and no added micronutrients, for 20 hours at 30 °C (starting OD_600_ = 0.03). Harvested cells were resuspended in 0.05 volumes of 50 mM NaP_i_, 300 mM NaCl, 20 mM imidazole, pH 7.5 supplemented with 0.1 mg mL^−1^ lysozyme and were lysed by sonication on ice for 5 minutes with 40% amplitude and 30% duty cycle. Lysate was clarified by centrifugation at 18 000*g* for 10 min at 4 °C, then loaded onto a 5 mL Histrap crude FF column (GE Healthcare). Protein was washed with 10 CV of binding buffer then eluted with a gradient from 20–500 mM imidazole. Protein-bearing fractions were concentrated using a 10 kDa MWCO Amicon centrifugal concentrator, then purified over Superdex 200 into 20 mM Na-MOPS pH 7.0. The heart-cut of the major elution peak was pooled and concentrated to ∼50 mg mL^−1^ using a 10 kDa MWCO Amicon centrifugal concentrator, and frozen at −70 °C. Protein was quantified spectrophotometrically using a calculated *ε*_280_ of 120 780 M^−1^ cm^−1^.

The gene encoding *Bt*Man2A was expressed, and *Bt*Man2A was purified using similar methods to Tailford *et al.*^4^ Briefly, an 8 ml starter culture of pET28a-*Bt*Man2A-His6 was used to inoculate 800 mL Luria Broth with 50 μg ml^−1^ of kanamycin. Expression cultures were grown at 37 °C with shaking at 180 rpm for about 3 hours until an OD 600 of 0.7 was reached. Isopropyl β-d-1-thiogalactopyranoside was added to 1 mM, and the temperature was then decreased to 16 °C and the culture incubated for an additional 21 h. The expression culture was harvested by centrifugation (5000*g*, 30 min, 4 °C) and the cell pellet was stored at −70 °C until purification. The cell pellet was resuspended in 40 ml of binding buffer, comprised of 50 mM HEPES, 10 mM imidazole, 0.5 M NaCl, 10 mM β-mercaptoethanol pH 8.0, with additional protease inhibitor (4-(2-aminoethyl)benzenesulfonyl fluoride, 1 mM) and 1 cOmplete™ Protease Inhibitor Cocktail tablet (Roche). Cells were lysed by sonication, the lysate was clarified by centrifugation at 15 000*g* for 30 min at 4 °C, and the supernatant was filtered through a 0.22 μM syringe filter and loaded onto a 1 mL HisTrap FF column (GE Healthcare). The protein was washed with 20 CV of binding buffer, then eluted with a gradient into buffer with 500 mM imidazole over 20 CV. Protein-bearing fractions were concentrated using a 30 kDa MWCO Vivaspin centrifugal concentrator, then purified over Superdex 200 into 50 mM HEPES pH 8.0, 150 mM NaCl, 2 mM DTT. The major elution peak fractions were pooled and buffer-exchanged into 50 mM HEPES pH 8.0, 2 mM DTT, and then loaded onto a 1 ml HiTrap Q column, equilibrated with the same buffer. The protein was eluted using a gradient into loading buffer with 0.5 M NaCl over 20 CV. The main peak fractions were buffer-exchanged into 10 mM HEPES pH 8.1, 5 mM TCEP, concentrated to 15 mg ml^−1^ and flash frozen with liquid nitrogen until use. Protein concentrations were determined spectrophotometrically using a calculated *ε*_280_ of 183 955 M^−1^ cm^−1^.

### Enzyme labelling detected by intact mass spectrometry and activity measurement

*Bs*GH164 and *Bt*Man2A were diluted to 0.2 mg mL^−1^ in assay buffer 1 (80 mM Na-MES pH 5.5, 160 mM NaCl). CmMan5A was diluted in assay buffer 2 (20 mM Na-MOPS pH 7.5). Inhibitors 1, 2 or 3 (10 mM, 1 μL), or 1 μL of water were mixed with 9 μL of each enzyme stock and the reactions were incubated at 30 °C with a heated lid to prevent evaporation. A 2 μL sample taken after 25 hours was diluted with 8 μL of 1% formic acid, 10% acetonitrile and analyzed as described previously.^20^ A separate 2 μL sample was taken at the same time and diluted with 23 μL of assay buffer 1 (*Bs*GH164), 398 μL of assay buffer 1 (*Bt*Man2A) or 23 μL of assay buffer 2 (CmMan5A). To each of these, in a single well of a black plastic 384-well plate, was added 25 μL of 0.2 mM 4-methylumbelliferyl β-d-mannopyranoside (Sigma) in water. Fluorescence was monitored continuously over 15 minutes *via* excitation at 360 nm and emission monitoring at 450 nm using a Clariostar (BMG Labtech) plate reader with temperature control set to 25 °C. Fluorescence over time measurements were converted into rates using a 4MU calibration series in each assay buffer and divided by the measured uninhibited control reaction rate to obtain residual activity.

### Enzyme crystallization

Crystals of *Bs*GH164 were grown in MRC maxi 48-well-plates using the sitting-drop vapor-diffusion method at 20 °C. The protein solution contained His_6_-*Bs*GH164 at 30 mg ml^−1^ in 20 mM HEPES pH 7.4 buffer. Well solution contained 100 mM ammonium tartrate dibasic (pH 7.0) and 13% (w/v) PEG 3350 and the protein : well solution ratio was 500 : 500 nl. To obtain an inhibitor-enzyme complex structure the inhibitor (1 mM final concentration) was added to the crystallization drop and incubated for 3 days. The crystals were subsequently cryo-protected in well solution containing 25% ethylene glycol and flash cooled in liquid nitrogen.

Crystals of *Cm*Man5A were initially grown from 30 mg mL^−1^ protein as described previously.^2^ A seed stock was prepared from needle clusters using the seed bead kit (Hampton research). *Cm*Man5A labelled with compound 3 was prepared by mixing 20 μL of 50 mg mL^−1^ enzyme with 5 μL of 10 mM compound 3 in 10% DMSO and incubating this for 25 hours at 30 °C. 100 nL of this was sampled and diluted into 10 μL of 1% formic acid, 10% acetonitrile and analyzed as above to assess the degree of labelling. Crystals of *Cm*Man5A labelled with compound 3 were grown in maxi 48-well-plates using the sitting-drop vapor-diffusion method at 20 °C by mixing 100 nL of seed stock into 600 nL of well solution, then adding 900 nL of protein solution. Crystals were apparent after overnight incubation but were allowed to continue growing for 3 days prior to freezing without cryo-protection.

*Bt*Man2A was crystallized in MRC maxi 48-well-plates using the sitting-drop vapour-diffusion method at 20 °C. The drop comprised 0.65 μl protein (at 10 mg ml^−1^ in 10 mM HEPES pH 8.1, 5 mM TCEP) and 0.35 μl mother liquor consisting of 14% (w/v) PEG 3350, 0.2 M sodium bromide, 0.1 M MES (2-(*N*-morpholino) ethanesulfonic acid) pH 6.7, for the crystal soaked with 2. The drop comprised 1.2 μl protein (at 7.5 mg ml^−1^ in 10 mM HEPES pH 8.1, 2.5 mM TCEP) and 1.2 μl mother liquor consisting of 12% (w/v) PEG 3350, 0.2 M sodium bromide, 0.1 M MES pH 5.5, for the crystal soaked with 3. The crystal soaks were performed with 10 mM 2 for 4 days, and with 1 mM 3, 1% (v/v) DMSO, for 3 days, and the crystals were fished into liquid nitrogen using a nylon CryoLoop (Hampton Research) *via* a cryoprotectant consisting of the mother liquor components with 25% ethylene glycol.

### Diffraction, structure solution, and refinement

All diffraction data were collected using a wavelength of 0.9763 Å. Diffraction data for *Bt*Man2A soaked with compounds 2 and 3 were collected at 100 K out to 2.05 and 1.85 Å, on beamlines I04-1 and I03, respectively. The data were processed using the Xia2 ^41^ pipeline with Dials.^42^ Diffraction data for CmMan5A in complex with compound 3 were collected out to 1.30 Å at 100 K on beamline I03 at Diamond Light Source (Harwell) and processed using the Xia2 pipeline with XDS.^43^ Diffraction data for *Bs*GH164 soaked with compounds 1 and 2 were collected out to 1.75 Å and 2.05 Å at 100 K on beamline I03 at Diamond Light Source (Harwell) and processed using the Xia2 pipeline with Dials (see ESI Table 1† for X-ray data collection and refinement statistics). Structures were solved by molecular replacement using Phaser^44^ or Molrep^45^ with the known unliganded enzyme structures (PDBID: 2JE8 (*Bt*Man2A), 1UUQ (*Cm*Man5A), 6T5O (*Bs*GH164)) as search models. The resulting solutions showed difference density for the bound ligands within the enzyme active sites. Ligand coordinates and dictionaries were generated using AceDRG^46^ or jLigand^47^ and built into the model using Coot,^48^ followed by alternating rounds of manual model building and density refinement using Coot and REFMAC^49^ within the CCP4 suite.^50^ Models were validated using MolProbity^51^ and the wwPDB OneDep validation server.

### Activity assay and ABPP of human plasma

5 mL of blood was collected from a healthy volunteer into an EDTA-containing collection tube. Cells were removed by centrifugation and plasma was aspirated gently. 450 μL of plasma was pH-adjusted with 50 μL of 500 mM pH 5.5 sodium citrate buffer. 50 μL of the buffered plasma was then diluted with 950 μL of ultrapure water. A negative control sample was prepared by supplementing diluted plasma with 0.05 volumes of 10% SDS and heating to 95 °C for 5 minutes. An additional 0.05 volumes of water was added to the diluted plasma for equivalence.

For activity measurements, 20 μL of each sample was mixed with 20 μL of 200 μM 4MU-β-mannoside (Sigma) in a 384-well plate and incubated at 22 °C for 30 minutes. 10 μL of 1 M Na_2_CO_3_ was then added and F360/450 was measured using a Clariostar plate reader (BMG Labtech). Free [4MU] was quantified against a calibration series prepared in 200 mM Na_2_CO_3_.

For ABPP, buffered and diluted plasma samples were supplemented with probe to a final concentration of 3 μM and incubated at 37 °C for 2 hours. 10 μL (∼4 μg total protein) was separated by SDS-PAGE and the resulting gel was imaged for fluorescence using a Typhoon 5 laser scanner (GE Healthcare).

### Fluorescence ABPP of mouse kidney extract

Kidneys from wild type mice were homogenized in KP_i_ buffer (25 mM K_2_HO_4_/KH_2_PO_4_, 0.1% (v/v) Triton X-100, protease inhibitor cocktail (Roche, EDTA-free)) with 1 mm glass beads and a FastPrep-24 homogenizer (MP Biomedicals). The basic labeling condition consists of equilibrating 25 μg total protein from the homogenate in McIlvaine buffer (150 mM, various pH) in a total volume of 10 μL, and ABP labeling with 5 μL of 3× concentrated ABP prepared in McIlvaine buffer. For concentration dependent labeling, mouse tissue homogenates were equilibrated in McIlvaine buffer pH 5.5 for 15 min at 37 °C, followed by incubation with various probe concentrations up to 10 μM 4 (end concentration, pH 5.5) at 37 °C for 1 h. For labeling at varying pH, mouse tissue homogenates were equilibrated in McIlvaine buffer pH 4.0–7.0 for 15 min at 37 °C, followed by incubating with 1 μM 4 (prepared in McIlvaine buffer pH 4.0–7.0) for 1 h at 37 °C. For labeling at varying incubation time, mouse kidney homogenates were prepared as above, and incubated with 1 μM 4 (end concentration; pH 5.5) at 37 °C for 2 min to 2 h. For cABPP, mouse tissue homogenates were pre-incubated with either SDS (2% (w/v)), cyclophellitol (3 μM), ABP MDW941 (3 μM), ABP JJB75 (3 μM), 1–3 (50 μM), 5 (3 μM), or 6 (50 μM) at 37 °C for 2 h, followed by incubation with 4 (3 μM) at 37 °C for 2 h. After incubation, samples were denatured with 3.75 μL 5× Laemmli's sample buffer at 98 °C for 5 min, resolved in 7.5% SDS-PAGE gels, and wet slab gels were scanned for Cy5^+^ fluorescence using a Typhoon FLA 9500 imager (GE Healthcare). Coomassie staining was carried out to confirm equal loading.

### Pull-down and LC/MS analysis with ABP 6

4.0 mg total protein from mouse kidney homogenates were incubated with either DMSO, 10 μM biotin probe 6, or 5 μM BODIPY-FL probe 5 followed by 10 μM 6, each step being incubated for 1 h at 37 °C in a total volume of 500 μL McIlvaine buffer pH 5.5 (75 mM citric acid/Na_2_HPO_4_). Samples were subsequently denatured with the addition of 125 μL 10% (w/v) SDS and boiling for 5 min at 100 °C. From here on, samples were prepared for pull-down with streptavidin beads as published earlier.^52^ After the pull-down procedure, half of the samples were treated by the trypsin digestion buffer (100 mM Tris-HCl pH 7.8, 100 mM NaCl, 1 mM CaCl_2_, 2% (v/v) acetonitrile (ACN) and 10 ng μL^−1^ trypsin) and the bead suspension was incubated in a thermoshaker at 37 °C overnight. The supernatant containing the trypsin-digested peptides was desalted using stage tips, followed by evaporation and redissolving in 75 μL sample solution (H_2_O/ACN/formic acid, 95/3/0.1, v/v/v). The beads containing active-site peptides were eluted with elution buffer (5% (v/v) formic acid, 25% (v/v) ACN, 70% (v/v) H_2_O, 10 μM biotin) for 30 min at 37 °C, after which the supernatant was desalted using stage tips and prepared for LC/MS analysis using the abovementioned procedures.

For the LC/MS analysis, 1 μL of sample was injected with phase A (0.1% (v/v) formic acid in ultrapure H_2_O) on a C18 column (Acquity UPLC M-Class 300 μm × 50 mm, packed with BEH C18 material of 1.7 μm diameter and 300 Å pore size particles), eluted with a 50 min gradient of 10% to 60% phase B (0.1% (v/v) formic acid in ACN), followed by 10 min equilibration to 1% phase B at a flow of 0.4 μL min^−1^, linked with electro-spray ionization (ESI) *via* Nano-spray source with ESI emitters (New Objectives) fused silica tubing 360 μm OD × 25 μm ID tapered to 5 ± 0.5 μm (5 nL cm^−1^ void volume) to a Synapt G2Si mass spectrometer (Waters) operating with Masslynx for acquisition and Ent3 software for polymer envelope signal deconvolution. The following settings in positive resolution mode were used: source temperature of 80 °C, capillary voltage 4.5 kV, nano flow gas of 0.25 Bar, purge gas 250 L h^−1^, trap gas flow 2.0 mL min^−1^, cone gas 100 L h^−1^, sampling cone 25 V, source offset 25, trap CE 32 V, scan time 3.0 s, mass range 400–2400 *m*/*z*. Lock mass acquisition was done with a mixture of Leu Enk (556.2771) and Glu Fib (785.84265), lockspray voltage 3.5 kV; Glufib fragmentation was used as calibrant. The PLGS (Waters) program was used for data analysis, protein ID or extraction of mgf files for further Mascot (Matrix Science) search analysis.

### ABPP of HEK293T cells overexpressing GBA2

HEK293T cells were purchased from ATCC and handled according to the published methods.^53^ 18.9 μg of lysates from GBA2-overexpressing HEK293T cells^54^ were pre-incubated with 1 μM of **JJB75** compound 1, 3, 5, 6, or 2% (w/v) SDS (with 5 min boiling at 98 °C when pre-incubation was completed) at pH 5.5 for 1 h at 37 °C, and next incubated with 1 μM ABP 4 at pH 5.5 for 2 h at 37 °C. Samples were denatured and subjected to SDS-PAGE and fluorescence detection. The gel was stained with Coomassie Brilliant Blue G250 to confirm equal protein loading.

## Author contributions

This study was conceived by NGSM, GJD, JMFGA, and HSO. NGSM, GJD, and HSO co-wrote the manuscript with input from all authors. TJMB, and C-SW synthesized all compounds under supervision of JDCC and HSO. NGSM, WAO, and ZA prepared recombinant protein samples, grew protein crystals, and solved protein complex structures. NGSM measured enzyme activity and intact MS. C-LK prepared biological samples and performed ABPP experiments under the supervision of JMFGA, and BIF collected proteomic data.

## Conflicts of interest

There are no conflicts to declare.

## Supplementary Material

OB-020-D1OB02287C-s001
