# Supplementary material for: Synthesis of broad-specificity activity-based probes for exo-β-mannosidases
Source: Org Biomol Chem. 2021 Dec 23;20(4):877–86. doi: 10.1039/d1ob02287c (PMC8790593; doi:10.1039/d1ob02287c)
Supplement: OB-020-D1OB02287C-s001 [file OB-020-D1OB02287C-s001.pdf]

Electronic Supplementary Material (ESI) for Organic & Biomolecular Chemistry.
This journal is © The Royal Society of Chemistry 2021

Supplemental Materials
for
Synthesis of Broad-Specificity Activity-Based Probes for Exo-β-Mannosidases

Nicholas G. S. McGregor[a], Chi-Lin Kuo[b], Thomas J. M. Beenakker[b], Chun-Sing Wong[b], Wendy A. Offen[a], Zachary Armstrong[a], Bogdan I. Florea[b], Jeroen D. C. Codée[b], Herman S. Overkleeft[b], Johannes M. F. G. Aerts[c,*], Gideon J. Davies[a,*]

[a]York Structural Biology Laboratory, Department of Chemistry, The University of York, Heslington, York, YO10 5DD, [b]Leiden Institute of Chemistry, Leiden University, Einsteinweg 55, 2300 RA Leiden, The Netherland, [c]Department of Medical Biochemistry, Leiden Institute of Chemistry, Leiden University, P. O. Box 9502, 2300 RA Leiden, The Netherlands, *Corresponding author.

Supplemental Synthetic Methods and Compound Characterization

All synthetic reagents were of a commercial grade and were used as received unless stated otherwise. Dichloromethane (DCM) and tetrahydrofuran (THF) were stored over 3 Å molecular sieves and *N,N*-dimethylformamide (DMF) was stored over 4 Å molecular sieves, which were dried *in vacuo* before use. All reactions were performed under an N_2 atmosphere unless stated otherwise. Reactions were monitored by analytical thin-layer chromatography (TLC) using Merck aluminum sheets pre-coated with silica gel 60 with detection by UV absorption (254 nm) and by spraying with a solution of $(NH_4)_6Mo_7O_{24}\cdot H_2O$ (25 g/L) and $(NH_4)_4Ce(SO_4)_4\cdot H_2O$ (10 g/L) in 10% sulfuric acid followed by charring at ± 150 ˚C or by spraying with an aqueous solution of $KMnO_4$ (7%) and K_2CO_3 (2%) followed by charring at ~150°C. Column chromatography was performed manually using either Baker or Screening Device silica gel 60 (0.04 - 0.063 mm) or a Biotage Isolera™ flash purification system using silica gel cartridges (Screening devices SiliaSep HP, particle size 15-40 µm, 60Å) in the indicated solvents. ^1H NMR and ^{13}C NMR spectra were recorded on Bruker AV-400 (400/101 MHz), Bruker AV-500 (500/126 MHz), Bruker DMX-600 (600/150 MHz) or Bruker AV-III-HD-850 spectrometer in the given solvent. Chemical shifts are given in ppm relative to the chloroform residual solvent peak or tetramethylsilane (TMS, used in MeOD solvent) as internal standard. The following abbreviations are used to describe peak patterns when appropriate: s (singlet), d (doublet), dd (doublet of doublet), m (multiplet), br (broad). 2D NMR experiments (HSQC, COSY and NOESY) were carried out to assign protons and carbons of the new structures. High-resolution mass spectra (HRMS) of compounds were recorded with a LTQ Orbitrap (Thermo Finnigan).

β-2,3-*O*-dibenzyl-2-*epi*-cyclophellitol (9):

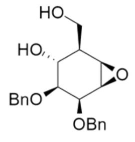

9

*m*CPBA (55%) (1.32 g, 4.2 mmol) was added to a solution of (1*S*,2*R*,5*S*,6*S*)-5,6-bis(benzyloxy)-2-(hydroxymethyl)cyclohex-3-enol[20] **7** (0.953 g, 2.8 mmol) in DCE (48 mL) and the mixture was heated to reflux. After complete conversion of the starting material the mixture was cooled to rt and silica was added to the mixture after which the solvents were removed *in vacuo*. The immobilized product was directly purified by column chromatography yielding benzylated β-manno cyclophellitol **9** (0.283 g, 0.794 mmol, 29%) and benzylated α-manno cyclophellitol **8** (0.180 g, 0.505 mmol, 18%) both as a white amorphous solid.

$[\alpha]_D^{22}$ + 64.7° (c = 1.0 DCM). ^1H NMR (600 MHz, $CDCl_3$) δ 7.45 – 7.41 (m, 2H), 7.39 – 7.27 (m, 8H), 4.85 (d, *J* = 12.1 Hz, 1H), 4.65 (d, *J* = 11.6 Hz, 1H), 4.63 (d, *J* = 12.1 Hz, 1H), 4.42 (d, *J* = 11.6 Hz, 1H), 4.10 – 4.05 (m, 2H), 3.93 (dd, *J* = 10.7, 5.1 Hz, 2H), 3.91 (t, *J* = 9.7 Hz, 1H), 3.28 (dd, *J* = 3.7, 2.0 Hz, 1H), 3.24 (dd, *J* = 4.9, 3.7 Hz, 1H), 3.20 (dd, *J* = 10.1, 5.0 Hz, 1H), 2.76 (s, 1H), 2.61 (s, 1H), 2.10 (m, 1H). ^{13}C NMR (151 MHz, CDCl3) δ 137.8, 137.5, 128.7, 128.6, 128.5, 128.2, 128.1, 79.7, 71.5, 71.4, 68.5, 67.1, 64.5, 54.6, 50.4, 44.8. HRMS: $[M+H]^+$ calculated for $C_{21}H_{25}O_5$ 357.16965, found 357.16965.

β-*manno*-configured cycloophellitol (1):

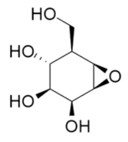

1

Benzylated β-*manno*-configured cyclophellitol **9** (41 mg, 0.115 mmol) was dissolved in 1,4-dioxane/^{t}BuOH (9:1) (2.5 mL) and purged with argon gas. Pd/C (10%) was added to this solution and the mixture was stirred under a H_2 atmosphere. After complete conversion of the starting material to the fully debenzylated product the mixture was filtered over a pad of celite and rinsed with H_2O. The filtrate was concentrated *in vacuo*. The product was crystallized in MeOH yielding β-*manno*-configured cyclophellitol **1** as a colourless crystalline solid (8.1 mg, 46 µmol, 40%).

mp 164°C. ^1H NMR (500 MHz, D_2O) δ 4.37 (t, *J* = 5.1 Hz, 1H), 3.99 (dd, *J* = 11.2, 4.2 Hz, 1H), 3.83 (dd, *J* = 11.2, 8.0 Hz, 1H), 3.56 (dd, *J* = 4.0, 1.9 Hz, 1H), 3.52 (dt, *J* = 8.3, 3.7 Hz, 2H), 3.46 (dd, *J* = 10.1, 8.9 Hz, 1H). ^{13}C NMR (126 MHz, D_2O) δ 72.3, 65.9, 65.3, 60.8, 56.1, 53.6, 44.1. HRMS: $[M+H]^+$ calculated for $C_7H_{13}O_5$ 177.07575, found 177.07576

(1*S*,2*R*,5*S*,6*S*)-5,6-bis(benzyloxy)-2-(methylthrichloroacetimidate)cyclohex-3-enol (10):

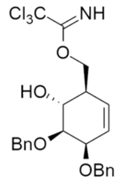

10

To a solution of *manno*-configured cyclohexene **9** (68.1 mg, 0.2 mmol) in DCM (4.25 mL) was added a 0.6 M solution of Cl_3CCN (0.5 mL, 0.3 mmol) in DCM and the mixture was cooled to 0°C. 0.4 M DBU (0.25 mL, 0.1 mmol) solution in DCM was added dropwise to the reaction mixture. The mixture was then allowed to reach rt and stirred for 30 min. The reaction mixture was diluted with DCM and silica was added. The solvents were removed *in vacuo* and the immobilized product was directly purified by column chromatography yielding cyclohexene imidate **10** as a colourless oil (75.4 mg, 0.16 mmol, 73%).

^1H NMR (400 MHz, $CDCl_3$) δ 8.31 (s, 1H), 7.39 – 7.26 (m, 10H), 5.95 – 5.84 (m, 2H), 4.74 (d, *J* = 11.7 Hz, 1H), 4.67 (d, *J* = 12.0 Hz, 1H), 4.64 (d, *J* = 12.0 Hz, 1H), 4.61 (dd, *J* = 6.5, 4.1 Hz, 1H), 4.56 (d, *J* = 11.7 Hz, 1H), 4.16 – 4.06 (m, 2H), 3.48 (dd, *J* = 10.1, 3.8 Hz, 1H), 2.87 (s, 1H), 2.62 (ddd, *J* = 8.3, 7.8, 3.5 Hz, 1H). ^{13}C NMR (100 MHz, $CDCl_3$) δ 163.0, 138.6, 138.0, 131.0, 128.6, 128.5, 128.1, 128.1, 128.0, 127.8, 125.8, 81.2, 71.8, 71.3, 69.5, 69.5, 66.8, 44.3. ESI-MS: $[M + Na]^+$ 508.0

(4a*R*,5*R*,6*S*,7*S*,8*S*,8a*R*)-6,7-bis(benzyloxy)-8-iodo-2-(trichloromethyl)-4a,5,6,7,8,8a-hexahydro-4*H*-benzo[*d*][1,3]oxazin-5-ol (11)

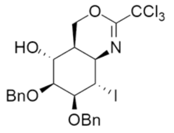

11

To a solution of cyclohexene imidate **10** (0.484 g, 1 mmol) in THF/H_2O (4:1) (25 mL) was added $NaHCO_3$ (0.294 g, 10 mmol), I_2 (0.888 g, 3.5 mmol) and the mixture was heated under reflux. After complete conversion of the starting material, the mixture was cooled to rt and diluted with EtOAc. 10% $Na_2S_2O_3$ (aq.) was added until the organic phase was colourless. The two phases were separated, and the aqueous phase was extracted with EtOAc (3x). The combined organic layers were washed with H_2O (3x), brine (3x), dried over $MgSO_4$, filtered, and concentrated *in vacuo*. Purification by column chromatography yielded iodo oxazine **11** as a yellow oil (0.459 g, 0.75 mmol, 75%).

^{1}H NMR (400 MHz, $CDCl_3$) *δ* 7.31 (ddq, *J* = 9.6, 6.9, 2.1 Hz, 10H), 5.05 (t, *J* = 2.4 Hz, 1H), 4.88 (dd, *J* = 11.2, 1.6 Hz, 1H), 4.79 (d, *J* = 12.3 Hz, 1H), 4.53 (d, *J* = 11.5 Hz, 1H), 4.45 (d, *J* = 12.3 Hz, 1H), 4.41 (d, *J* = 11.6 Hz, 1H), 4.19 (dd, *J* = 7.0, 2.6 Hz, 1H), 4.16 (dd, *J* = 5.6, 2.7 Hz, 1H), 4.13 – 4.11 (m, 1H), 4.10 (d, *J* = 2.7 Hz, 1H), 4.07 (q, *J* = 2.6 Hz, 1H), 2.63 (br s, 1H), 2.50 (m, 1H). ^{13}C NMR (100 MHz, $CDCl_3$) δ 151.6, 137.5, 128.7, 128.5, 128.4, 128.2, 128.1, 127.9, 79.3, 77.1, 71.8, 71.1, 67.2, 64.7, 59.3, 33.8, 26.9. ESI-MS: $[M + Na]^+$ 633.8. HRMS: $[M+H]^+$ calculated for $C_9H_{14}INO_4$ 328.00403, found 328.00409.

2,3-*O*-dibenzylated β-*manno*-configured cyclophellitol aziridine (12)

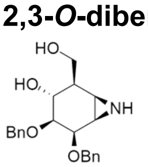

12

Iodo oxazine **11** (0.459 g, 0.75 mmol) was dissolved in a 1,4-dioxane/H_2O/AcOH (1:1:8) mixture (30 mL) and stirred overnight at rt. The mixture was concentrated *in vacuo*, co-evaporated with toluene (3x) and the residue was dissolved in MeOH (30 mL). To the solution was added $NaHCO_3$ (1.26 g, 15 mmol) and stirred overnight at rt. The solids were filtered over a pad of celite and the filtrate was concentrated *in vacuo*. The crude was dissolved in DCM and washed with H_2O (1x). The aqueous layer was extracted with DCM (5x) and the combined organic layers was dried over $MgSO_4$, filtered and concentrated *in vacuo*. Purification by column chromatography using neutral and activated silica followed by precipitation in cold Et_2O yielded benzylated aziridine **12** as a white powder (0.155 g, 0.437 mmol, 58%).

^{1}H NMR (400 MHz, CD_2Cl_2) *δ* 7.46 – 7.25 (m, 10H), 4.77 (d, *J* = 11.5 Hz, 1H), 4.62 (d, *J* = 11.5 Hz, 1H), 4.49 (d, *J* = 11.6 Hz, 1H), 4.43 (d, *J* = 11.5 Hz, 1H), 4.18 (t, *J* = 5.2 Hz, 1H), 3.92 (dd, *J* = 10.7, 5.7 Hz, 1H), 3.84 (dd, *J* = 10.8, 4.8 Hz, 1H), 3.77 (t, *J* = 9.8 Hz, 1H), 3.23 (dd, *J* = 10.1, 4.7 Hz, 1H), 2.82 (s, 2H), 2.42 – 2.29 (m, 2H), 2.05 (s, 1H). ^{13}C NMR (100 MHz, CD_2Cl_2) *δ* 138.6, 128.9, 128.9, 128.7, 128.5, 128.3, 81.2, 71.6, 71.5, 69.7, 66.1, 64.9, 44.7. HRMS: $[M+H]^+$ calculated for $C_{21}H_{26}NO_4$ 356.18563, found 356.18570.

β-*manno*-configured cycloophellitol aziridine (2)

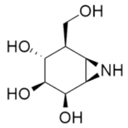

2

NH_3 gas was condensed at -60°C and liquid NH_3 was collected (~2.5 mL). To the liquid NH_3 was added lithium metal (16.5 mg, 2.5 mmol), upon addition of the lithium the solution turned dark blue. The mixture was stirred until all of the lithium was completely dissolved and a solution of benzylated aziridine **12** (35.5 mg, 0.1 mmol) in THF (2 mL) was added drop wise. The reaction was stirred for 30 min at -60°C and H_2O (1.5 mL) was added dropwise to the reaction mixture. The mixture was gradually warmed to rt and co-evaporated with H_2O (3x). The crude product was dissolved in H_2O and treated with Amberlite IR-120 NH_4^+ for 2 h. The resin was filtered, the filtrate was concentrated *in vacuo* and re-treated with Amberlite IR-120 NH_4^+ (3x). The product was dissolved in MeOH and precipitated in 0°C ether under vigorous stirring. The precipitate was filtered and dried over a stream of air yielding β-aziridine **2** as a white powder (17.4 mg, 0.1 mmol, quantitative).

^{1}H NMR (400 MHz, D_2O) *δ* 4.27 (t, *J* = 5.3 Hz, 1H), 3.89 (dd, *J* = 10.9, 4.5 Hz, 1H), 3.70 (dd, *J* = 10.9, 8.7 Hz, 1H), 3.44 (dd, *J* = 9.3, 4.9 Hz, 1H), 3.35 (t, *J* = 8.8 Hz, 1H), 2.66 – 2.52 (m, 2H), 2.01 (ddd, *J* = 8.3, 4.6, 3.6 Hz, 1H). ^{13}C NMR (100 MHz, D_2O) *δ* 73.4, 66.4, 65.5, 62.3, 43.6, 33.1, 32.6. HRMS: $[M+H]^+$ calculated for $C_7H_{13}NO_4$ 176.09173, found 176.09170.

N-octylazido β-*manno*-configured cycloophellitol aziridine (3)

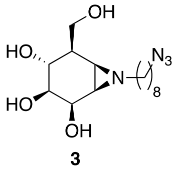

3

Cyclophellitol aziridine **2** (0.4 mmol), 1-azido-8-iodooctane[21] (0.6 mmol, 168 mg) and K_2CO_3 (237 mg, 1.72 mmol) in DMF (4 mL) was stirred at 80°C. After stirring overnight the reaction mixture was concentrated *in vacuo* and purification over silica gel column chromatography (6% MeOH in DCM → 8% MeOH in DCM) gave **3** (32.1 mg, 98 µmol, 64%) as a clear oil.

^{1}H NMR (400 MHz, MeOD) *δ* 4.14 (t, *J* = 3.8 Hz, 1H), 3.82 (dd, *J* = 10.2, 6.0 Hz, 1H), 3.70 (dd, *J* = 8.3, 11.7 Hz, 1H), 3.51 (dd, *J* = 6.7, 4.7 Hz, 1H), 3.45 (dd, *J* = 6.6, 4.1 Hz, 1H), 3.28 (t, *J* = 6.8 Hz, 2H), 2.42 – 2.33 (m, 1H), 2.23 – 2.13 (m, 1H), 2.10 – 2.05 (m, 2H), 2.05 – 1.96 (m, 1H), 1.58 (t, *J* = 6.9 Hz, 4H), 1.44 – 1.34 (m, 8H). ^{13}C NMR (101 MHz, MeOD) *δ* 103.8, 98.3, 93.8, 92.6, 89.6, 80.6, 73.0, 72.3, 71.3, 58.6, 58.6, 58.4, 58.1, 56.5, 55.9. HRMS: calculated for $[C_{15}H_{29}N_4O_4]^+$ 329.21833, found 329.21823.

Activity-based probes (ABPs) 4, 5 and 6

General Click Procedure: To a solution of aziridine-azide (1 equiv.) and desired alkyne (1 equiv.) in DMF (2.0 mL) copper(II)-sulfate pentahydrate (0.66 equiv., 1 M in H_2O) and sodium ascorbate (0.76 equiv., 1 M in H_2O) were added. After stirring overnight at room temperature, the reaction mixture was concentrated *in vacuo*, purified with HPLC-MS (linear gradient, A: 50 mM NH_4HCO_3 in H2O, B: MeCN, in 12 min) and lyophilized.

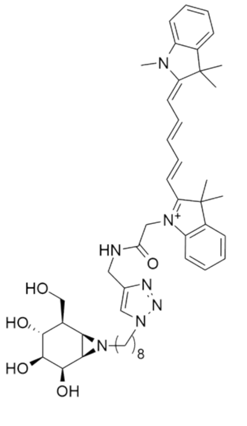

Cy5 4: Aziridine **3** (13.1 mg, 39.8 µmol) and Cy5-alkyne **7** (20 mg, 36 µmol) were treated following the General Click Procedure. Purification by HPLC (40% → 60%, A in B) gave ABP **4** (5.3 mg, 6.0 µmol, 17%) as a blue powder.

^{1}H NMR (850 MHz, MeOD) δ 8.25 (dt, J = 3.6, 2.0 Hz, 2H), 7.85 (s, 1H), 7.49 (d, J = 7.4 Hz, 2H), 7.44 – 7.39 (m, 2H), 7.32 – 7.24 (m, 4H), 6.62 (t, J = 12.4 Hz, 1H), 6.29 (d, 2.2 Hz, 1H), 6.27 (d, 2.2 Hz, 1H), 4.41 (s, 2H), 4.37 (t, J = 7.1 Hz, 2H), 4.14 (t, J = 4.1 Hz, 1H), 4.10 (d, J = 7.5 Hz, 1H), 4.09 (d, J = 7.9 Hz, 1H), 3.81 (dd, J = 10.2, 6.0 Hz, 1H), 3.68 (dd, J = 8.0, 9.1 Hz, 1H), 3.63 (s, 3H), 3.50 (dd, J = 6.5, 4.7 Hz, 1H), 3.45 (dd, J = 6.6, 4.2 Hz, 1H), 2.37 – 2.32 (m, 1H), 2.25 (t, J = 7.3 Hz, 2H), 2.18 – 2.13 (m, 1H), 2.08 – 2.02 (m, 2H), 2.01 – 1.98 (m, 1H), 1.87 – 1.85 (m, 2H), 1.82 – 1.79 (m, 2H), 1.79 – 1.70 (m, 14H), 1.58 – 1.52 (m, 2H), 1.49 – 1.42 (m, 2H), 1.35 – 1.28 (m, 8H). ^{13}C NMR (214 MHz, MeOD) δ 175.7, 175.4, 174.6, 155.6, 155.5, 144.2, 143.5, 142.6, 142.5, 129.8, 129.7, 126.6, 126.3, 126.2, 123.4, 123.2, 112.0, 111.9, 104.4, 104.2, 75.6, 70.1, 65.7, 64.4, 61.4, 51.3, 44.9, 44.8, 44.2, 43.2, 36.5, 35.6, 31.5, 31.3, 30.3, 30.3, 29.9, 28.2, 28.1, 27.9, 27.8, 27.4, 27.3, 26.4. LC/MS analysis: Rt 6.18 min (linear gradient 10→90% B in 12.5 min), m/z 848.27 $[M]^+$. HRMS: calculated for $[C_{50}H_{70}N_7O_5]^+$ 848.54329, found 848.54278.

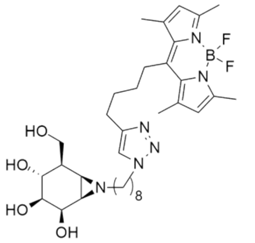

Bodipy-FL 5: Aziridine **3** (7.1 mg, 22 µmol) and BODIPY-FL alkyne (7.1 mg, 22 µmol) were treated following the General Click Procedure. Purification by HPLC (42% → 48%, A in B) gave ABP **5** (5.13 mg, 7.82 µmol, 36%) as an orange powder.

^{1}H NMR (600 MHz, MeOD) δ 7.74 (s, 1H), 6.11 (s, 2H), 4.35 (d, J = 7.2 Hz, 1H), 4.34 (d, J = 6.6 Hz, 1H), 4.13 (t, J = 4.1 Hz, 1H), 3.81 (dd, J = 10.2, 6.0 Hz, 1H), 3.68 (dd, J = 10.2, 8.4, 1H), 3.50 (dd, J = 6.7, 4.7 Hz, 1H), 3.47 – 3.42 (m, 1H), 3.35 (s, 3H), 3.32 – 3.28 (m, 2H), 3.04 – 2.97 (m, 2H), 2.78 (d, J = 7.2 Hz, 1H), 2.77 (d, J = 7.2 Hz, 1H), 2.44 (s, 6H), 2.38 (s, 6H), 2.36 – 2.30 (m, 1H), 2.14 (dt, J = 11.7, 7.1 Hz, 1H), 2.07 – 1.97 (m, 3H), 1.93 – 1.83 (m, 4H), 1.68 – 1.61 (m, 2H), 1.57 – 1.50 (m, 2H), 1.36 – 1.24 (m, 8H). 13C NMR (151 MHz, MeOD) δ 154.9, 148.5, 147.9, 142.2, 132.6, 123.4, 122.6, 75.6, 70.1, 65.7, 64.4, 61.4, 51.9, 49.9, 44.9, 44.1, 43.2, 32.2, 31.2, 30.8, 30.3, 30.3, 29.9, 29.1, 28.2, 27.3, 25.9, 16.5, 14.4. LC/MS analysis: Rt 6.15 min (linear gradient 10→90% B in 12.5 min), m/z 657.07 [M + H]+. HRMS: calculated for [C34H52BF2N6O4]+ 657.41117, found 657.41122.

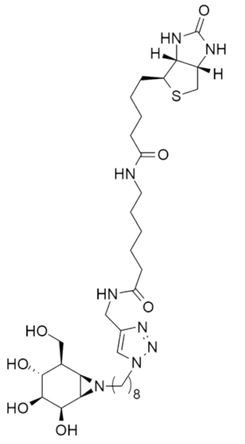

Biotin 6: Azidirine-azide **3** (9.1 mg, 28 µmol) and biotin alkyne (11.5 mg, 28 µmol) were treated following the General Click Procedure. Purification by HPLC (18% → 24%, A in B) gave ABP **6** (6.5 mg, 8.9 µmol, 32%) as a white powder.

^{1}H NMR (500 MHz, MeOD) δ 7.84 (s, 1H), 4.49 (dd, J = 7.8, 4.9 Hz, 1H), 4.42 (s, 1H), 4.38 (d, J = 7.6 Hz, 1H), 4.36 (d, J = 7.6 Hz, 1H), 4.30 (dd, J = 7.9, 4.4 Hz, 1H), 4.14 (t, J = 3.9 Hz, 1H), 3.81 (dd, J = 10.2, 5.9 Hz, 1H), 3.69 (dd, J = 10.1, 8.8 Hz, 1H), 3.54 – 3.43 (m, 2H), 3.24 – 3.18 (m, 1H), 3.17 – 1.12 (m, 2H), 2.92 (dd, J = 12.7, 5.0 Hz, 1H), 2.70 (d, J = 12.7 Hz, 1H), 2.39 – 2.32 (m, 1H), 2.26 – 2.13 (m, 5H), 2.10 – 2.03 (m, 2H), 2.03 – 1.96 (m, 1H), 1.90 (s, 5H), 1.79 – 1.70 (m, 1H), 1.65 – 1.53 (m, 6H) 1.52 – 148 (m, 2H), 1.43 – 1.39 (m, 2H), 1.36 – 1.25 (m, 9H). ^{13}C NMR (126 MHz, MeOD) δ 176.0, 175.9, 146.2, 124.1, 75.6, 70.1, 65.7, 64.4, 63.4, 61.6, 61.4, 57.0, 51.3, 44.9, 44.1, 43.2, 41.0, 40.2, 36.8, 36.7, 35.6, 31.2, 30.3, 30.3, 30.1, 29.9, 29.8, 29.5, 28.2, 27.5, 27.3, 26.9, 26.5. LC/MS analysis: Rt 3.98 min (linear gradient 10→90% B in 12.5 min), m/z 723.20 $[M+H]^+$. HRMS: calculated for $[C_{34}H_{59}N_8O_7S]^+$ 723.42219, found 723.42220.

NMR Spectra

^{1}H-NMR and ^{13}C-NMR spectra of **1** in D_2O

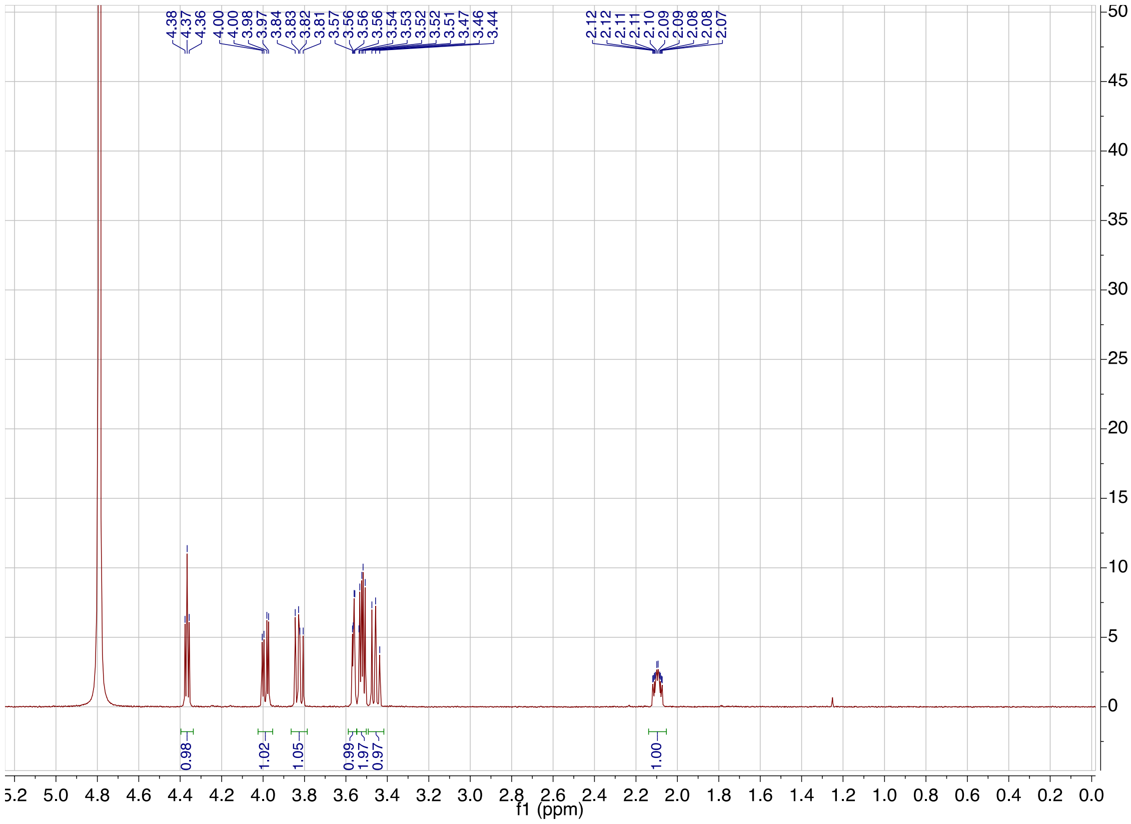

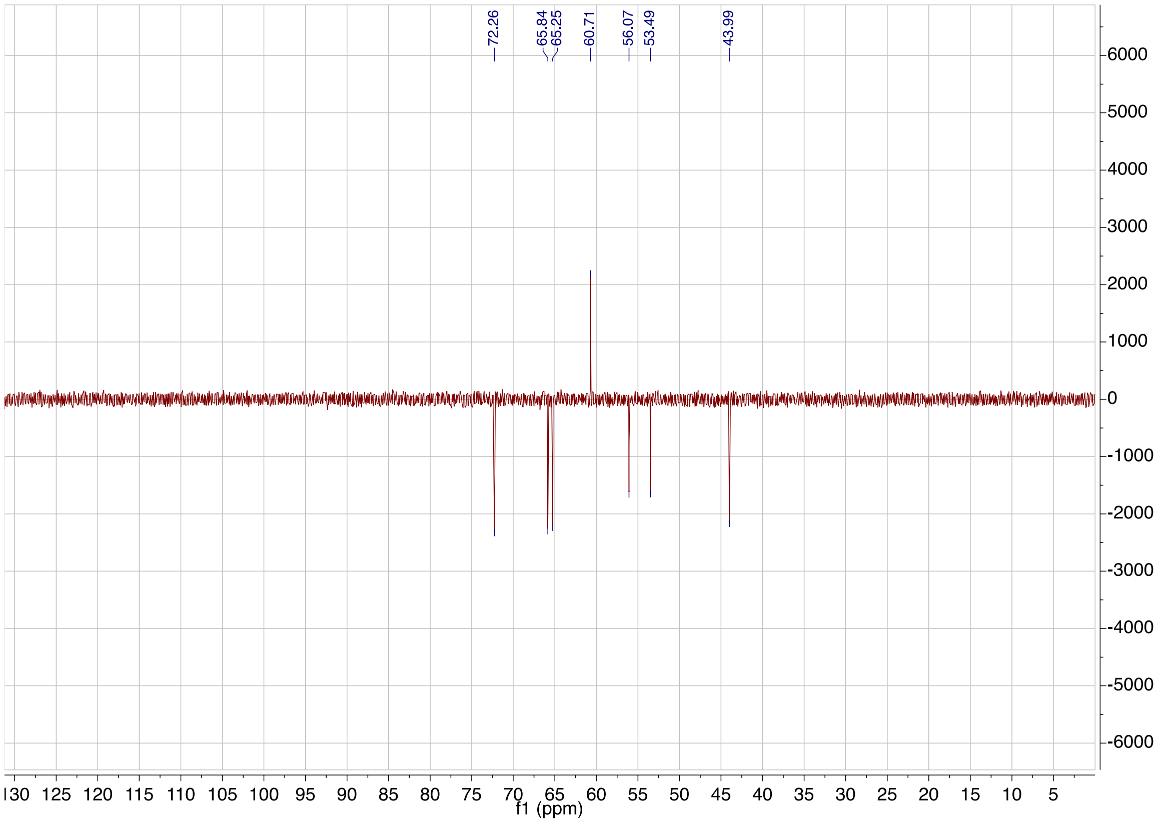

^{1}H-NMR and ^{13}C-NMR spectra of **2** in D_2O

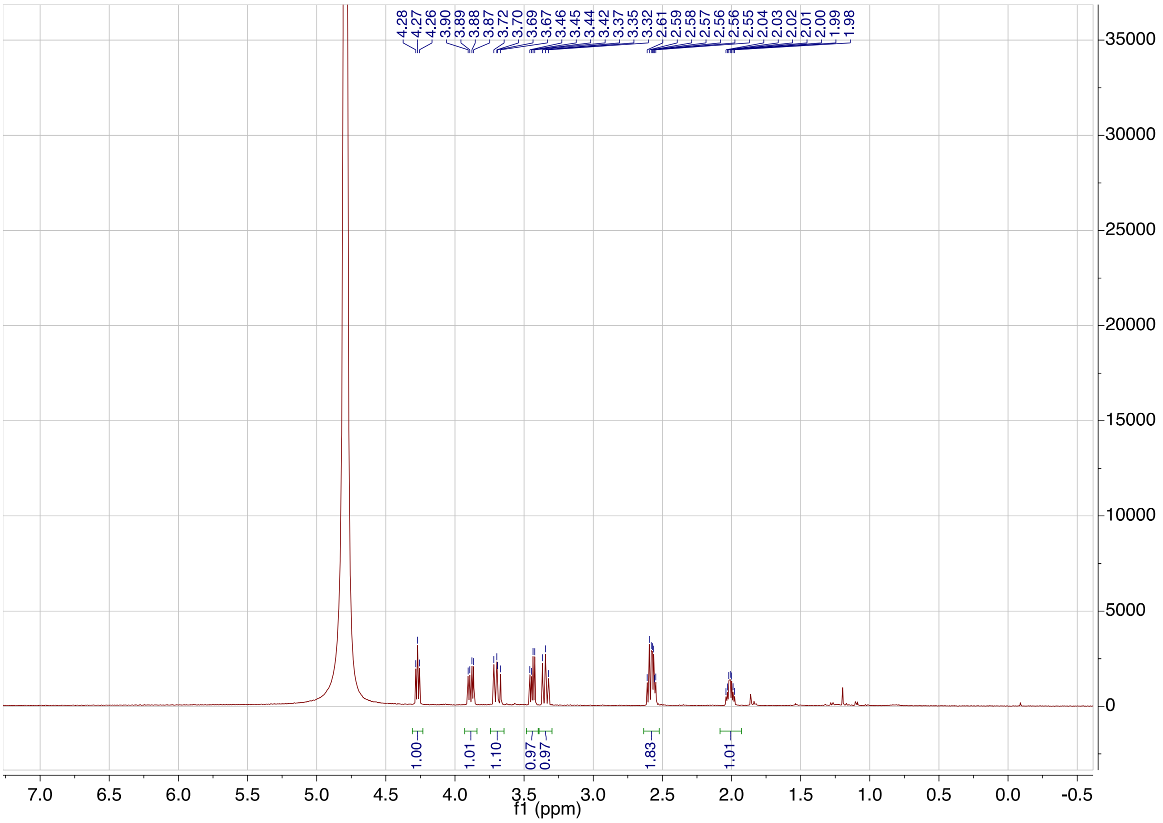

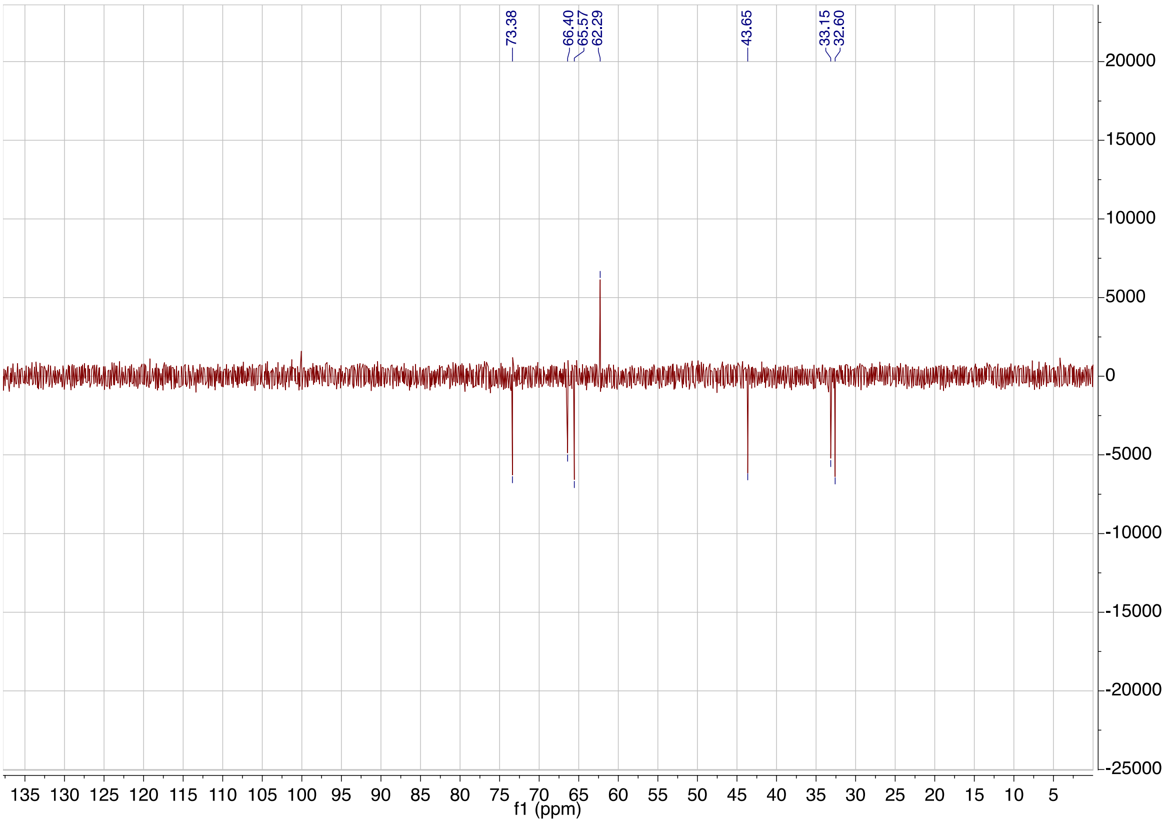

^{1}H-NMR and ^{13}C-NMR spectra of **3** in MeOD

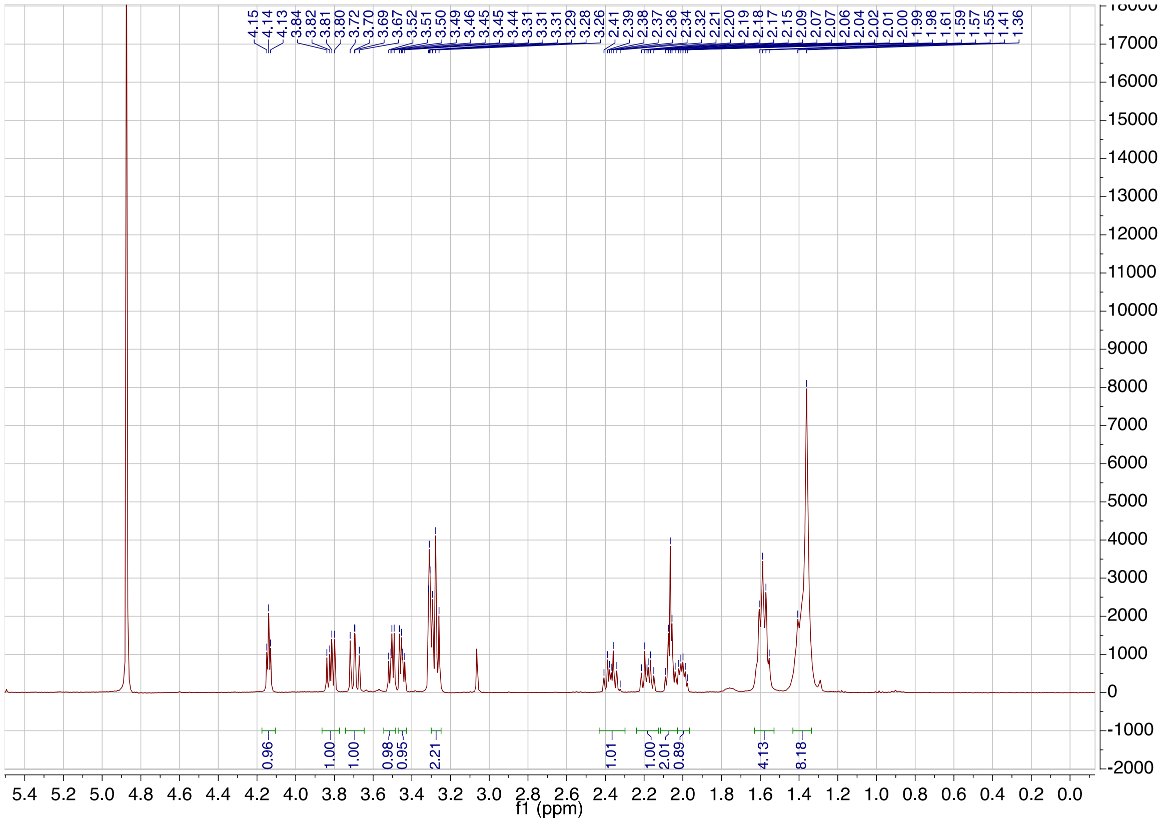

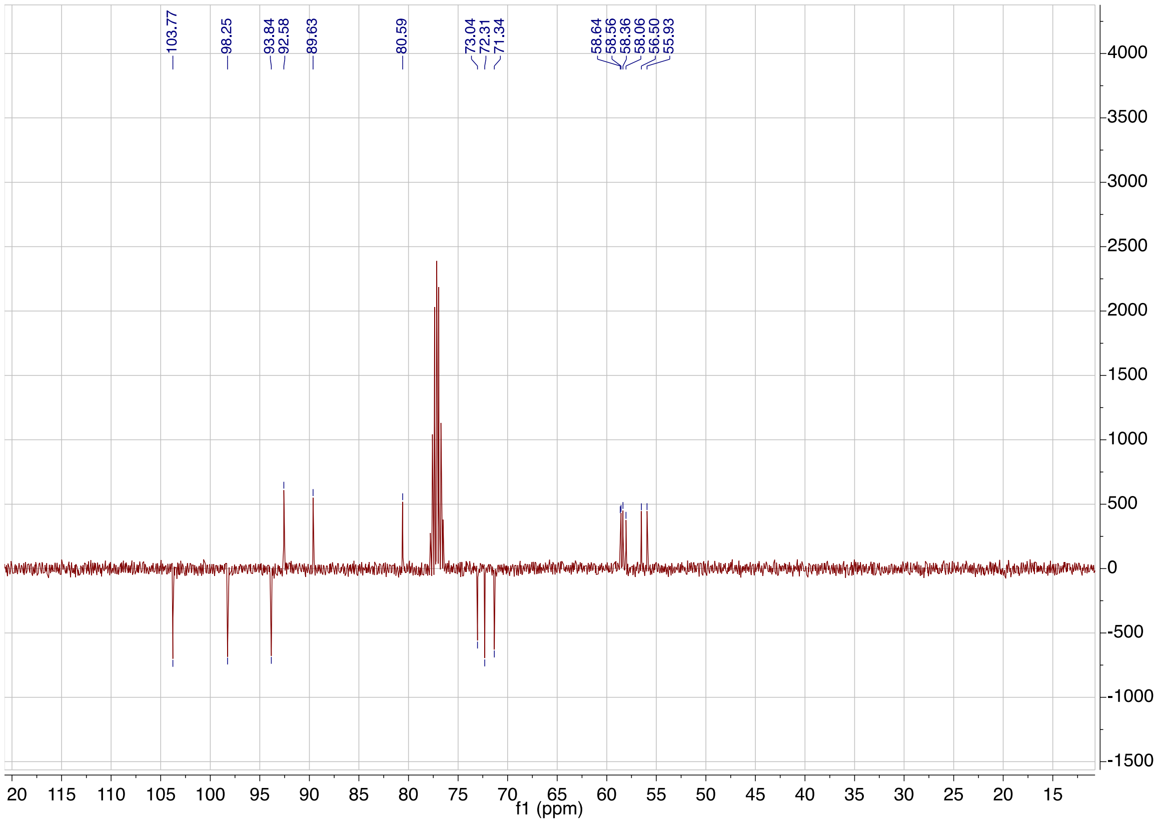

^{1}H-NMR and ^{13}C-NMR spectra of **4** in MeOD

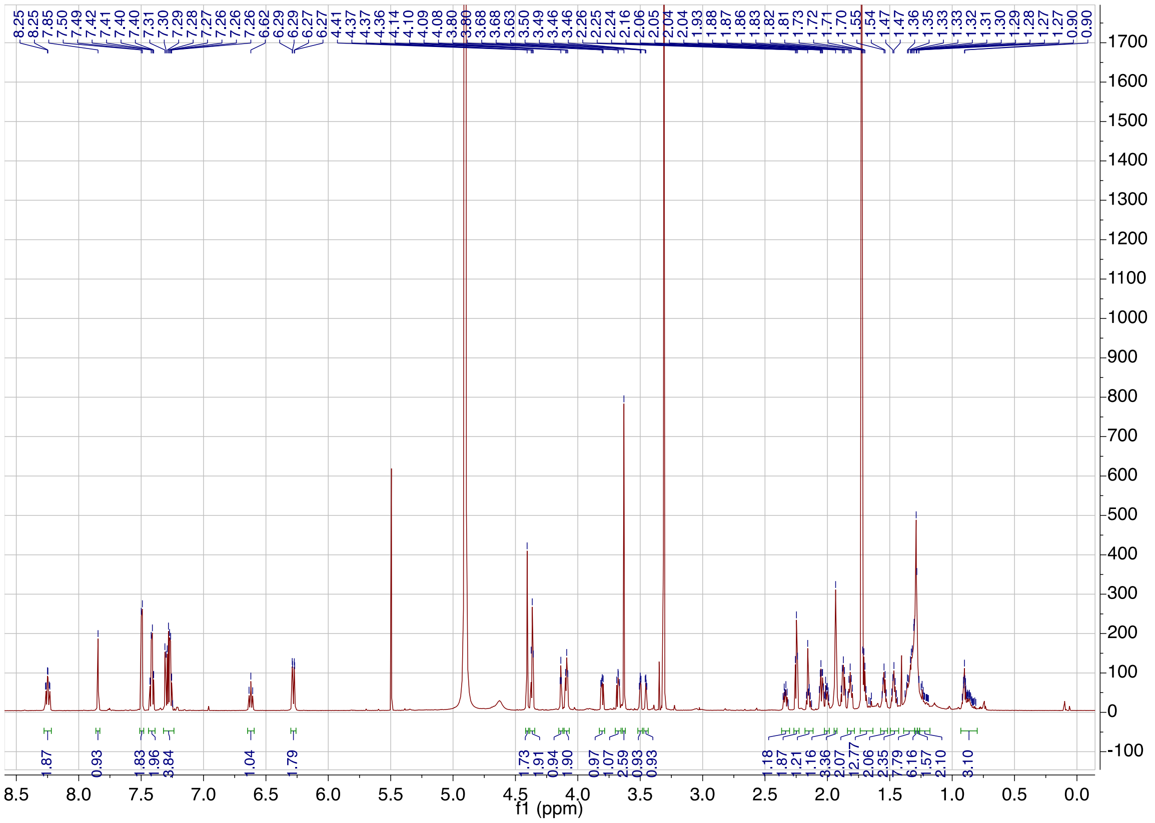

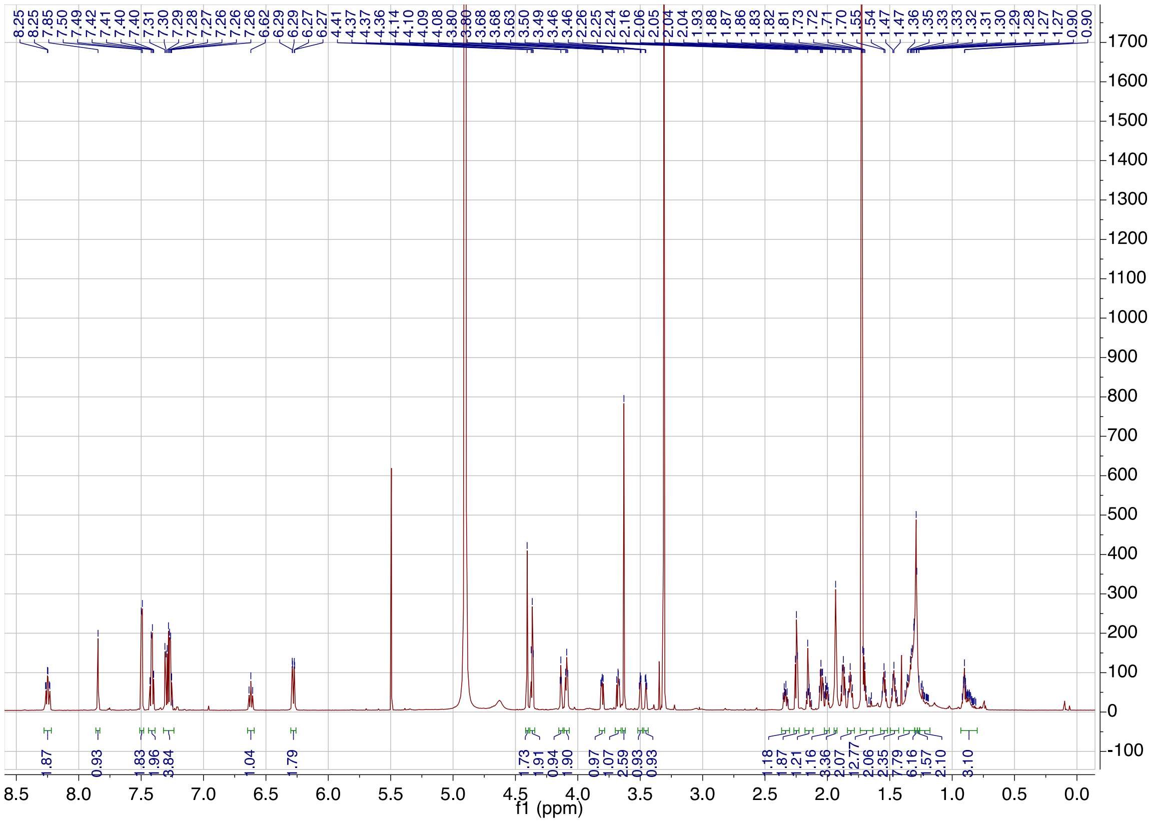

^{1}H-NMR and ^{13}C-NMR spectra of **5** in MeOD

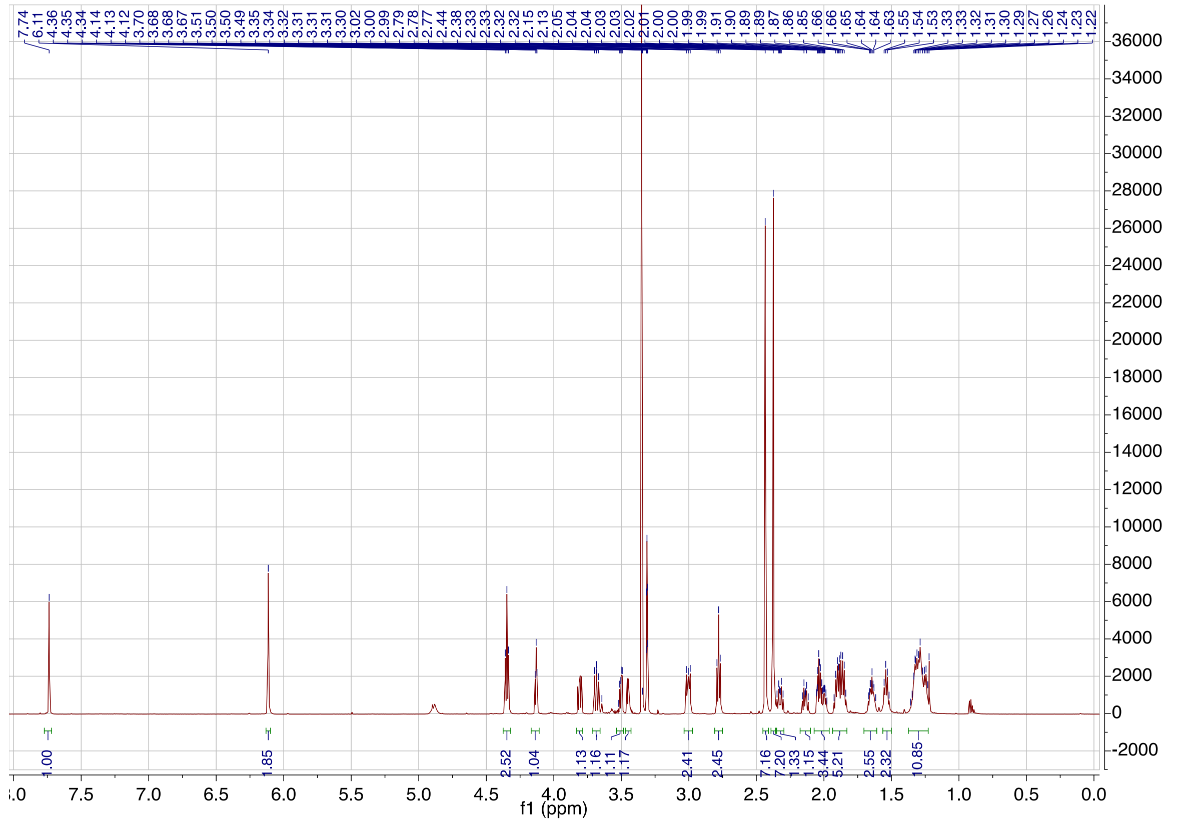

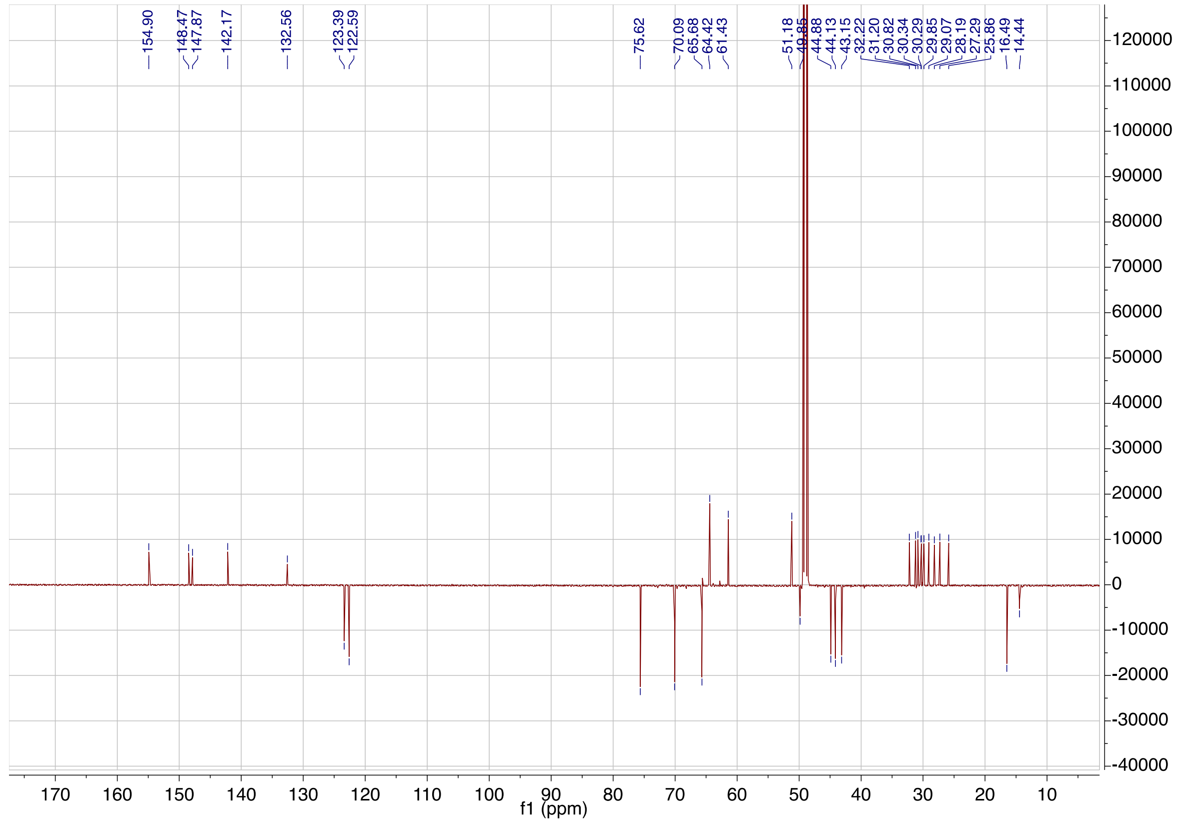

^{1}H-NMR and ^{13}C-NMR spectra of **6** in MeOD

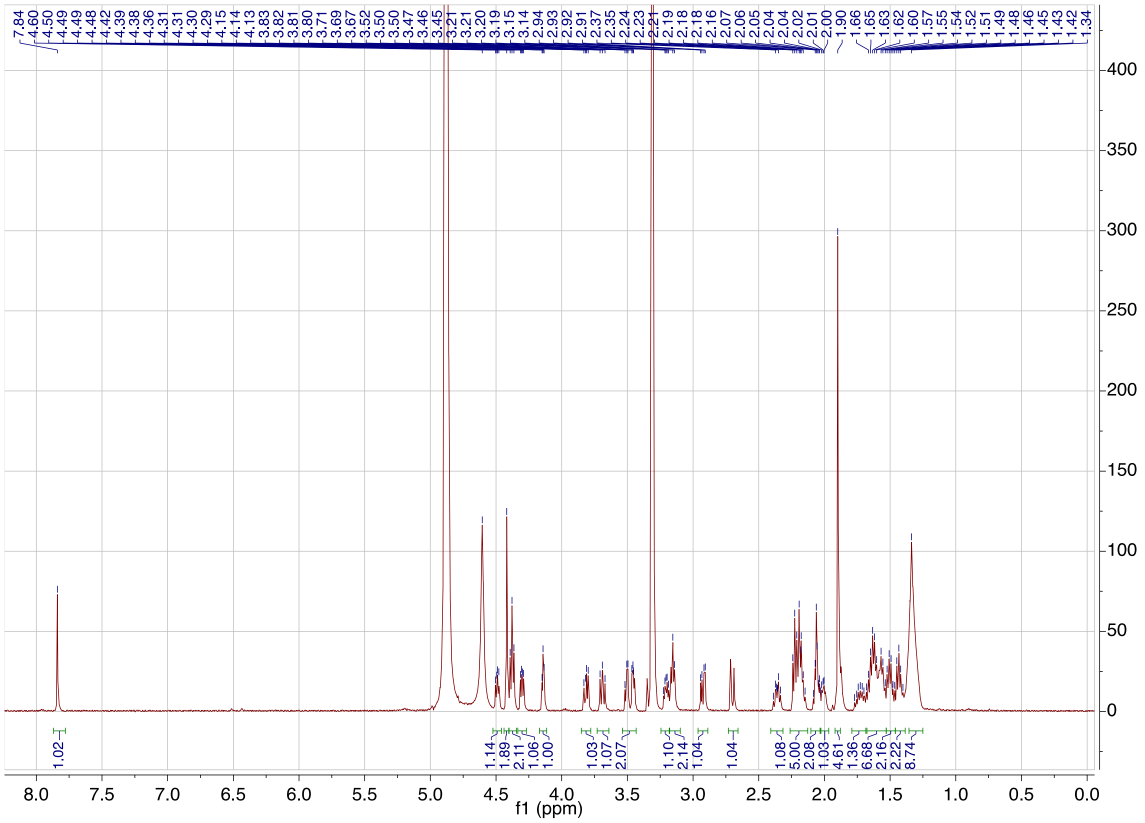

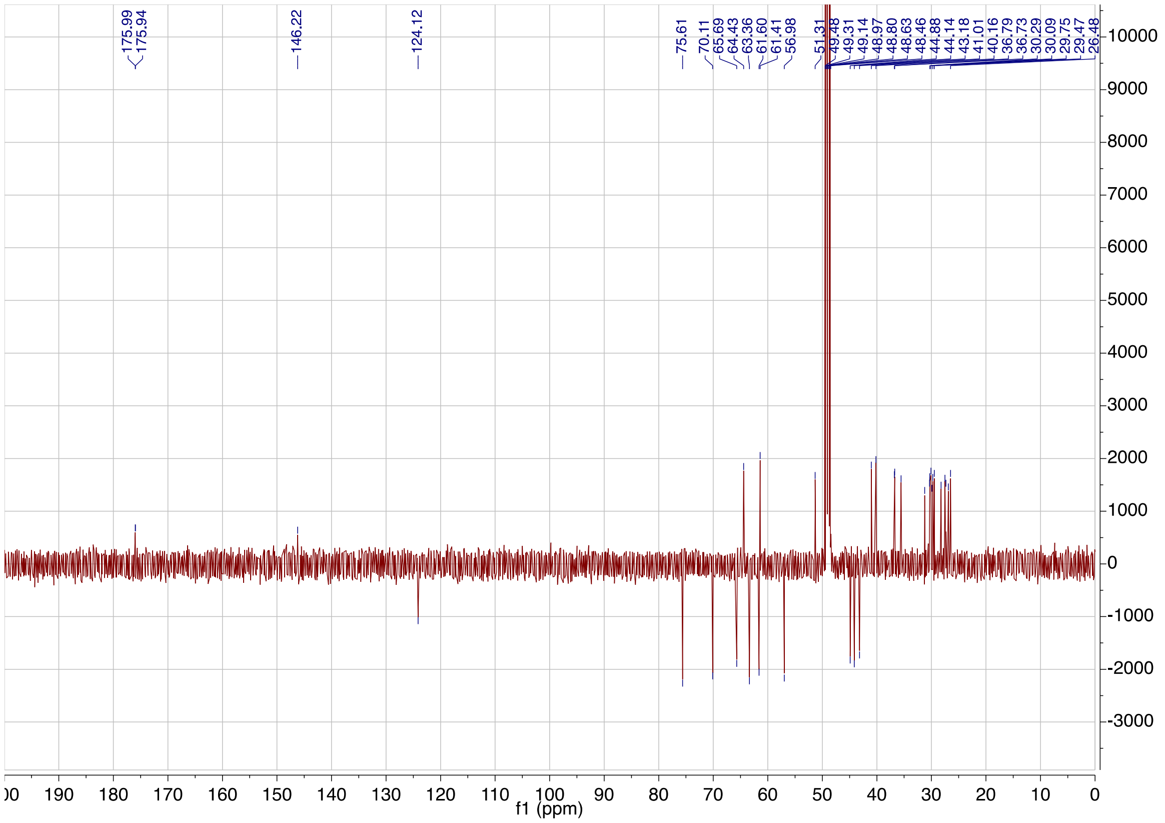

^{1}H-NMR and ^{13}C-NMR spectra of **9** in $CDCl_3$

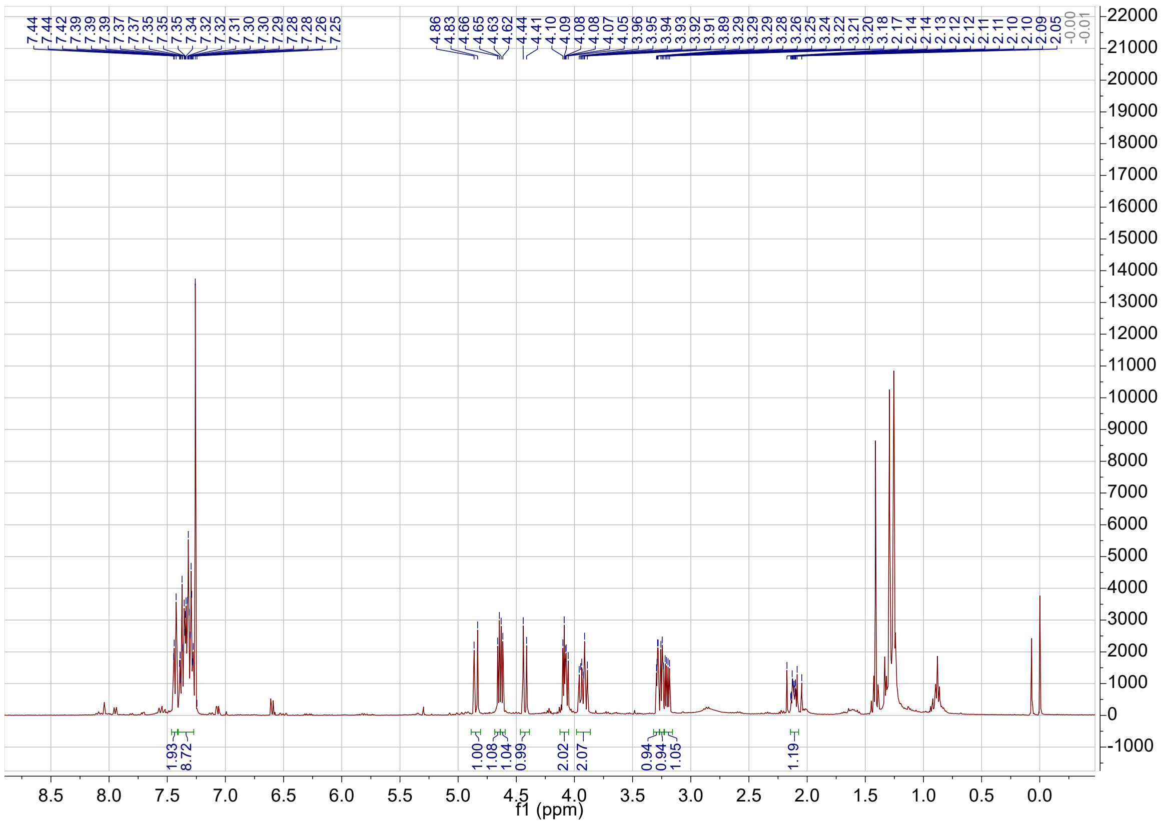

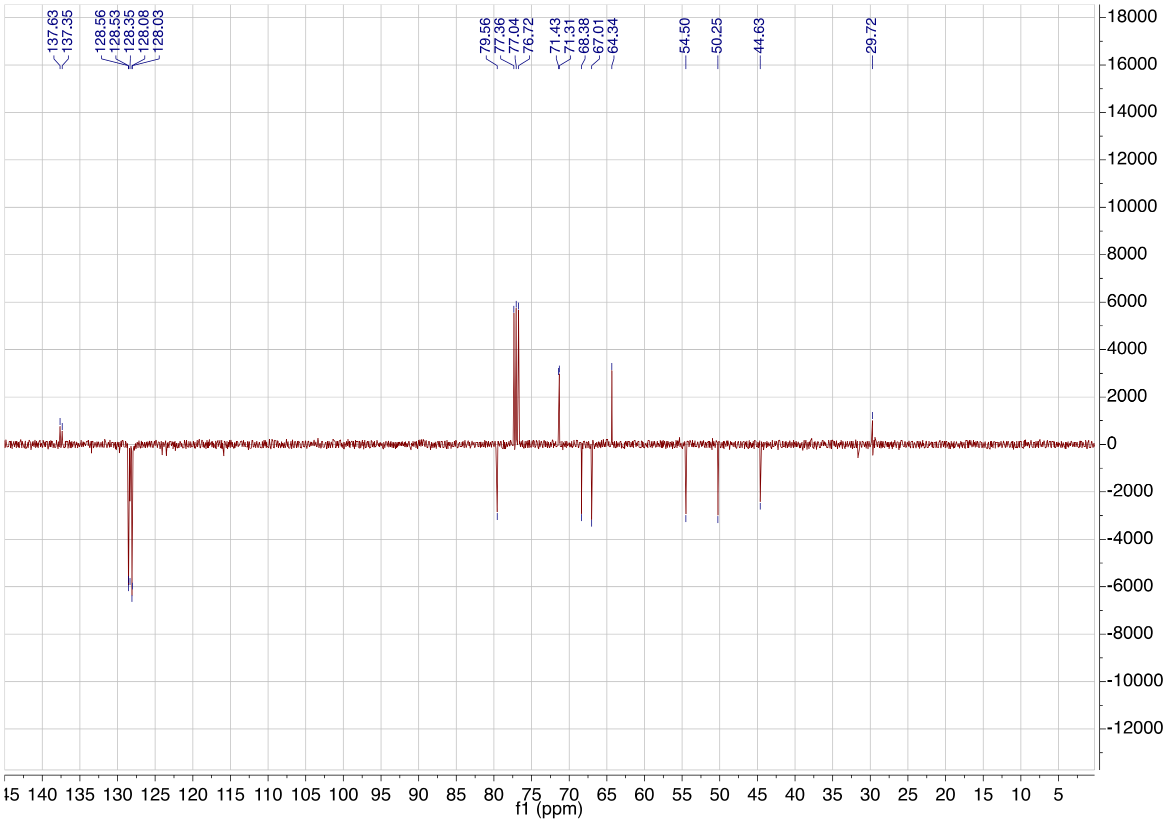

^{1}H-NMR and ^{13}C-NMR spectra of **10** in $CDCl_3$

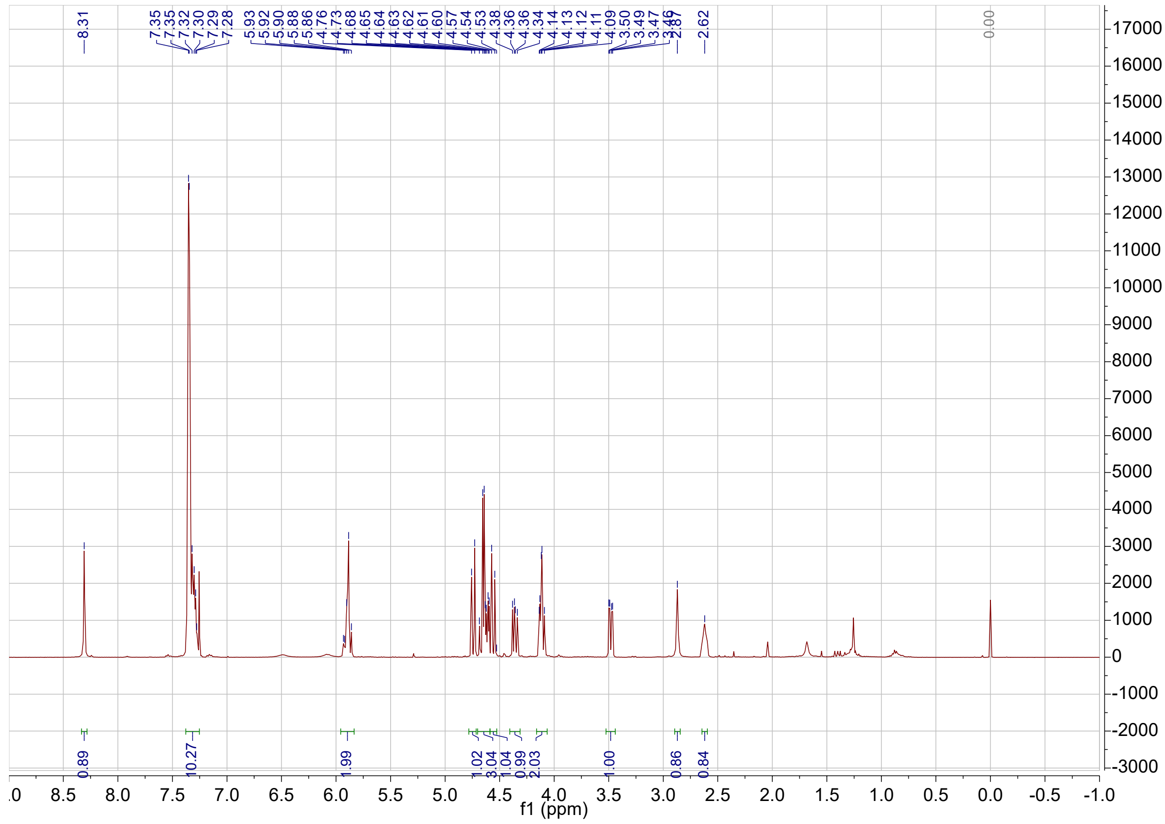

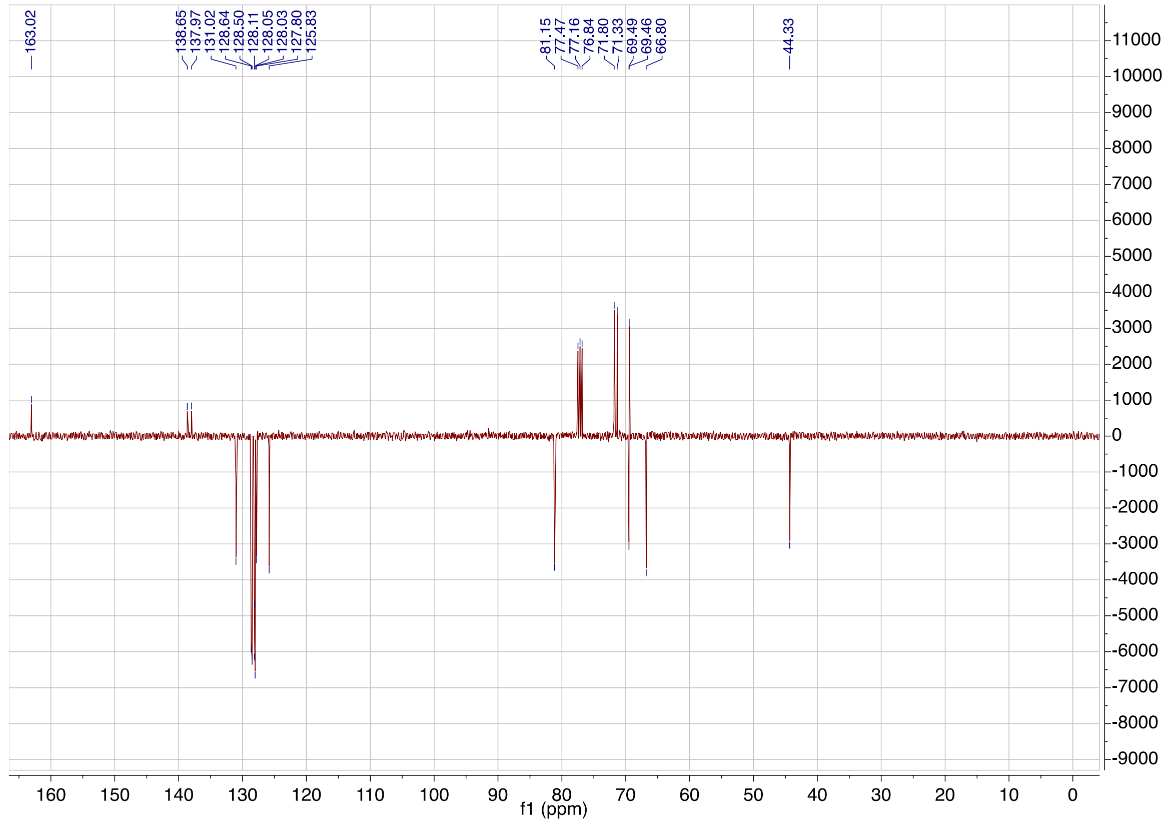

^{1}H-NMR and ^{13}C-NMR spectra of **11** in $CDCl_3$

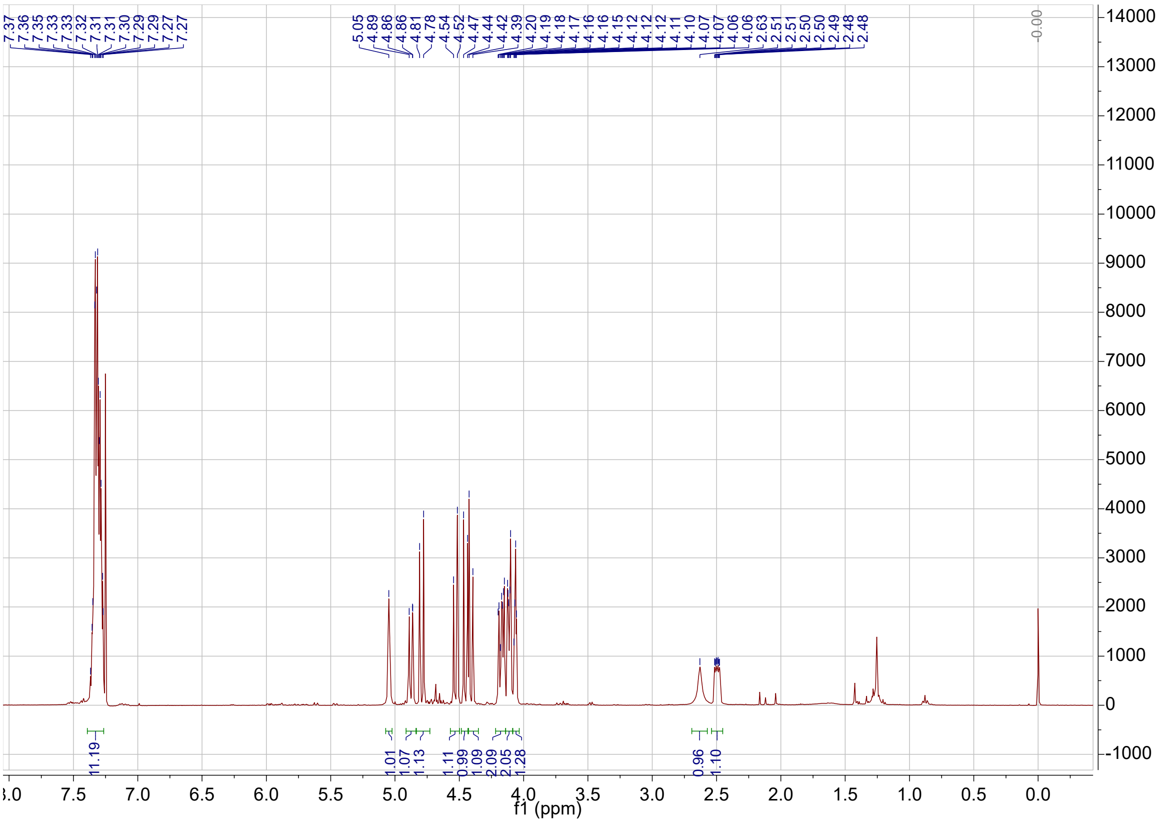

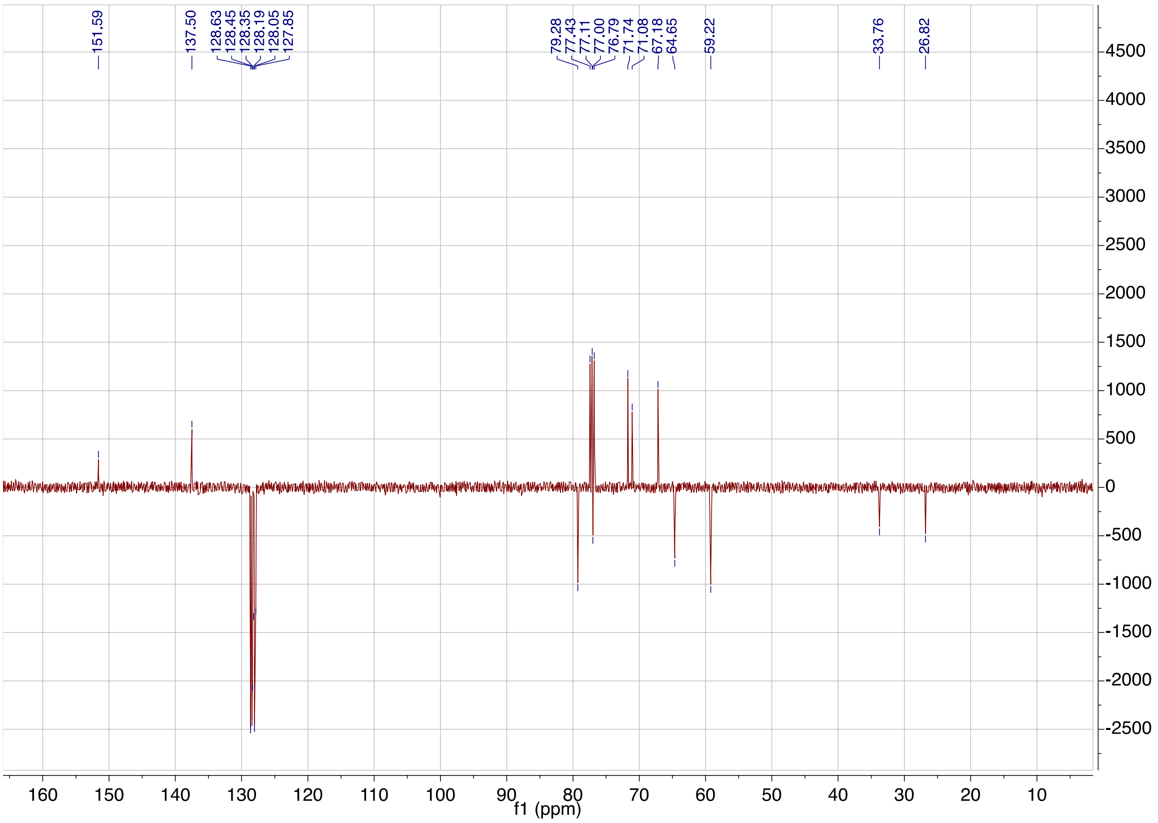

^{1}H-NMR and ^{13}C-NMR spectra of **12** in CD_2Cl_2

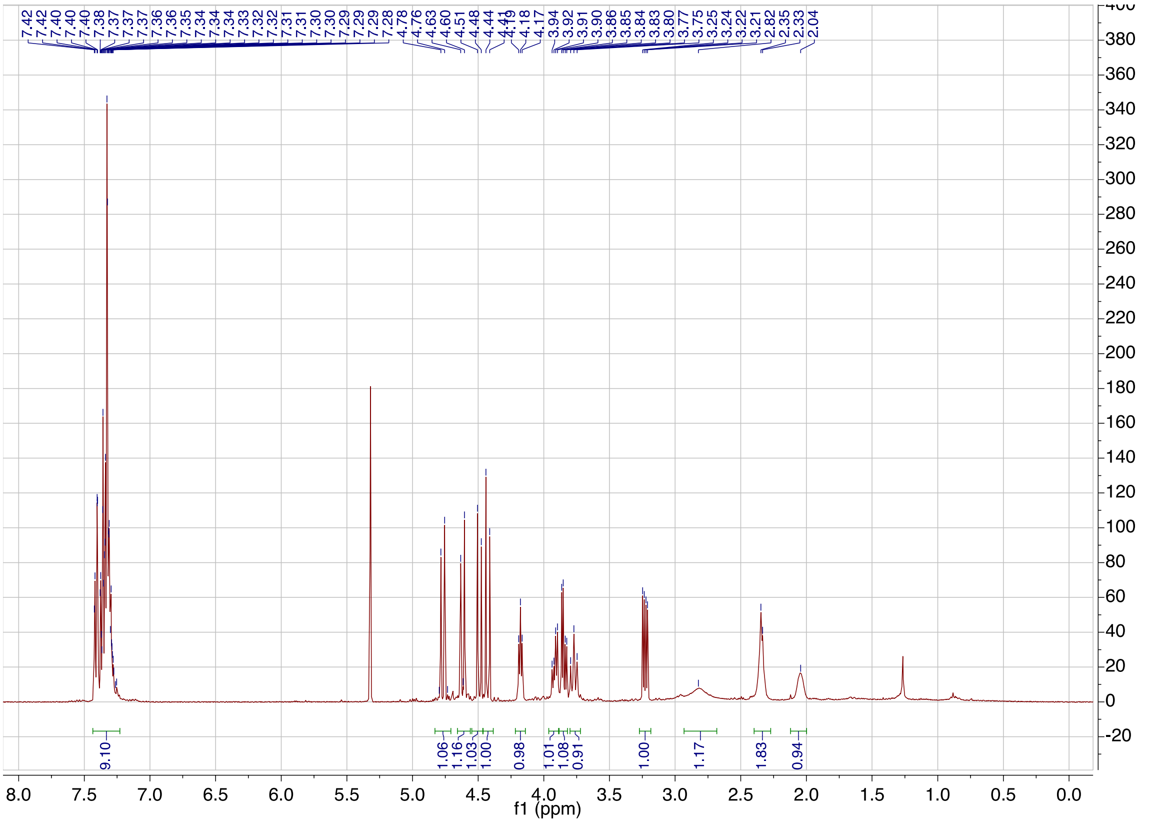

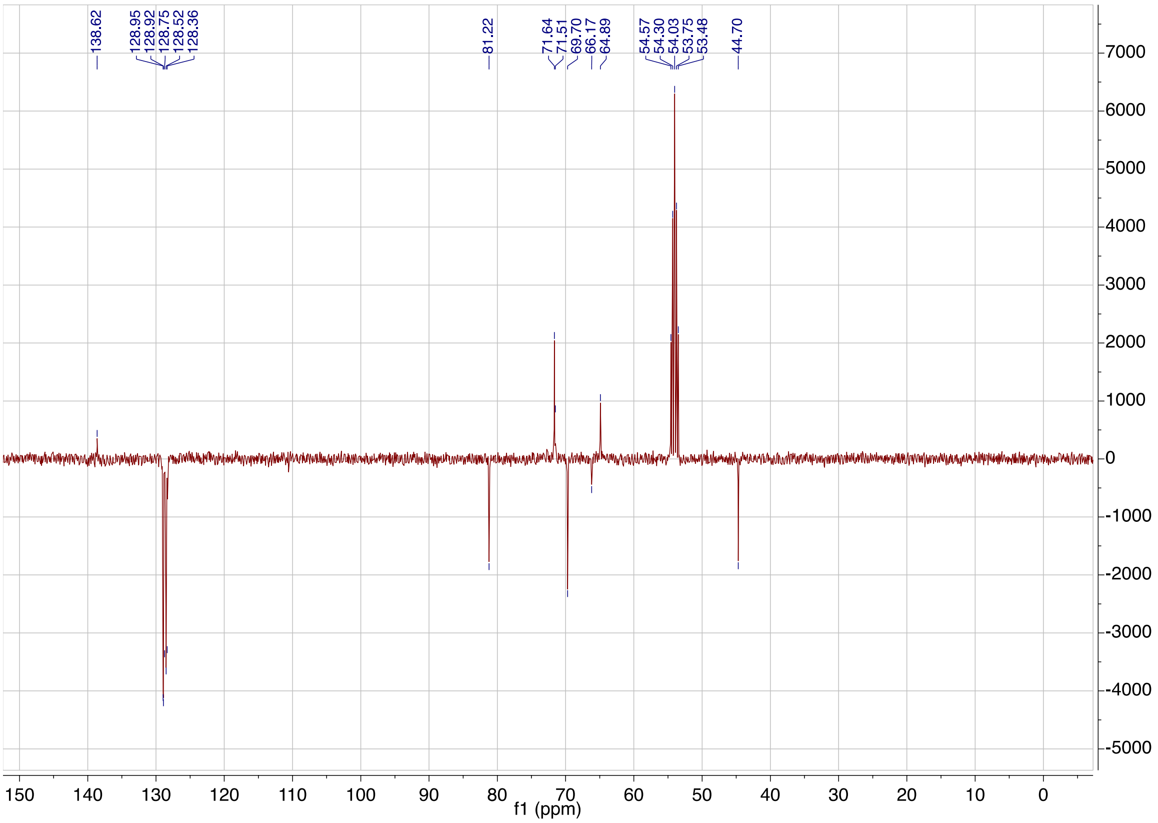

Supplemental Figures and Tables

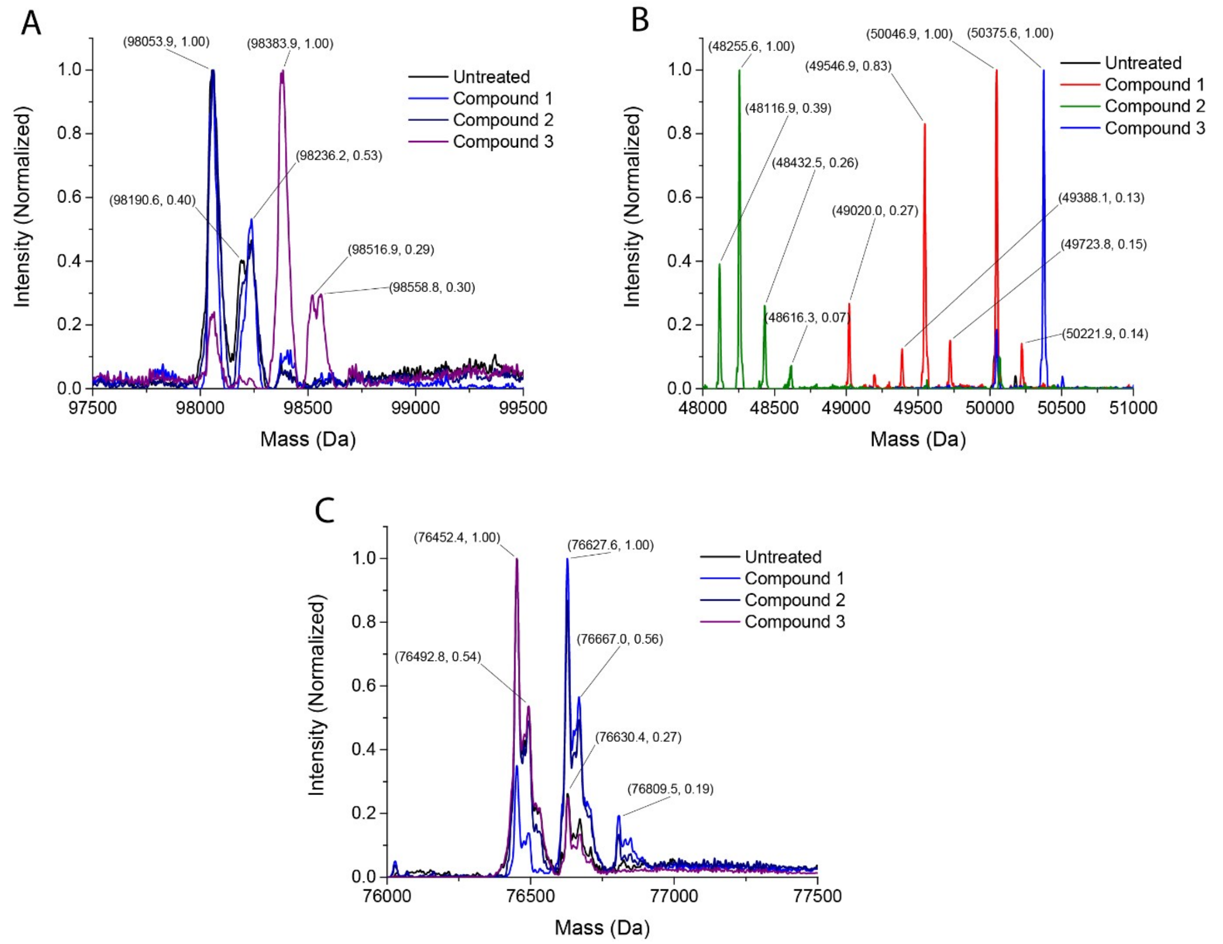

Supplemental figure 1. Intact MS of mannosidases following inhibitor treatments. A) *Bt*Man2A (expected mass = 98174 Da) before and after 25 hours treatment with each of the compounds shown at 1 mM. Δm(**1**) = +176 Da, Δm(**2**) = +175 Da, Δm(**3**) = +328 Da. The dominant peak at 98054 is attributed to N-terminal demethionation and the peak at ~98236 is attributed to subsequent gluconoylation, which would be indistinguishable from modification with **1** or **2**. The peak at ~89191 is attributed to the native sequence. B) *Cm*Man5A (expected mass = 50178.7 Da) before and after 25 hours treatment with each of the compounds shown at 1 mM. The single peak at 50046.9 is attributed to N-terminal demethionation. Peaks at 50221.9 and 49723.8 Da following treatment with **1** are attributed modification with compound **1**. Peaks at 49020.0 and 49546.9 Da are attributed to proteolytic removal of MVAESNSAVAPT or MVAESN from the N-terminus, respectively. The peaks at 48255.6 and 48116.9 are attributed to proteolytic removal of MVAESNSAVAPTANVA from the N-terminus and removal of HHH or HHHH from the C-terminus. The peak at 48432.5 Da attributed to subsequent addition of compound **2**. C) *Bs*164 (expected mass = 76584 Da) before and after 25 hours treatment with each of the compounds shown at 1 mM. The peak at 76452.4 is attributed to demethionation and the peak at 76630 is attributed to subsequent gluconoylation. The origin of the peaks at m+40 is unclear but may be attributed to either acetylation with small systematic mass error (expected +42) or an unknown combination of modifications. The increase in height and -3 Da shift of the peak at 76627.6 is attributed to labelling with compound **1** while the appearance of the peak at 76809.5 is attributed to labelling of the gluconoylated protein with compound **1**.

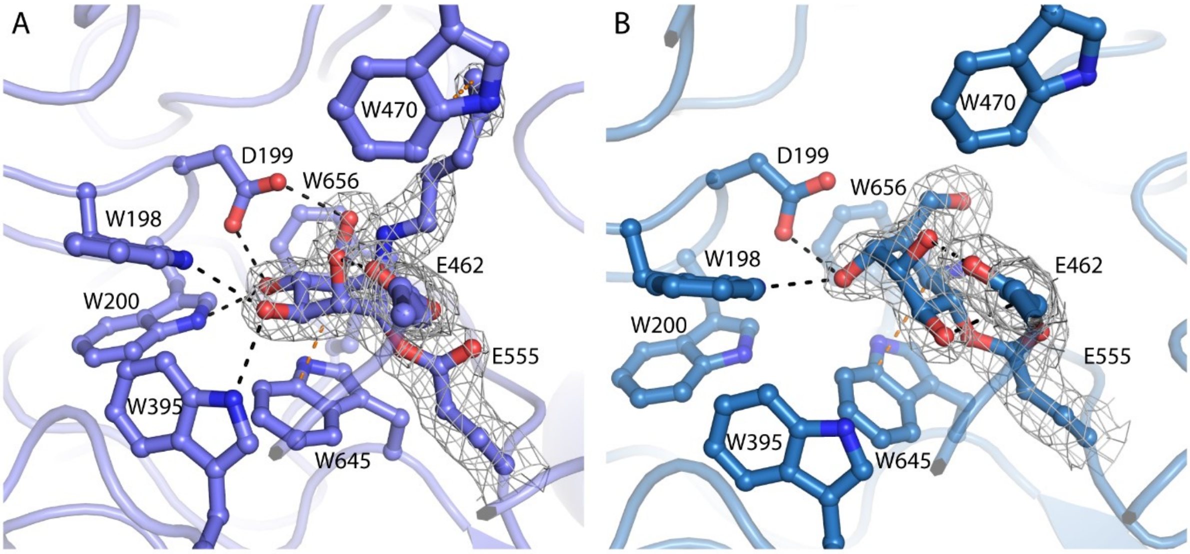

Supplemental figure 2. A) Structure of *Bt*Man2A labelled with **3**. 2F_o-F_c electron density, contoured to 1.0σ, is shown around the ligand and the catalytic residues. Apparent hydrogen bonding interactions are shown as black dashed lines while apparent hydrophobic close contacts are shown as orange dashed lines. B) Structure of *Bt*Man2A labelled with **2**. 2F_o-F_c electron density and interactions are shown as in panel A.

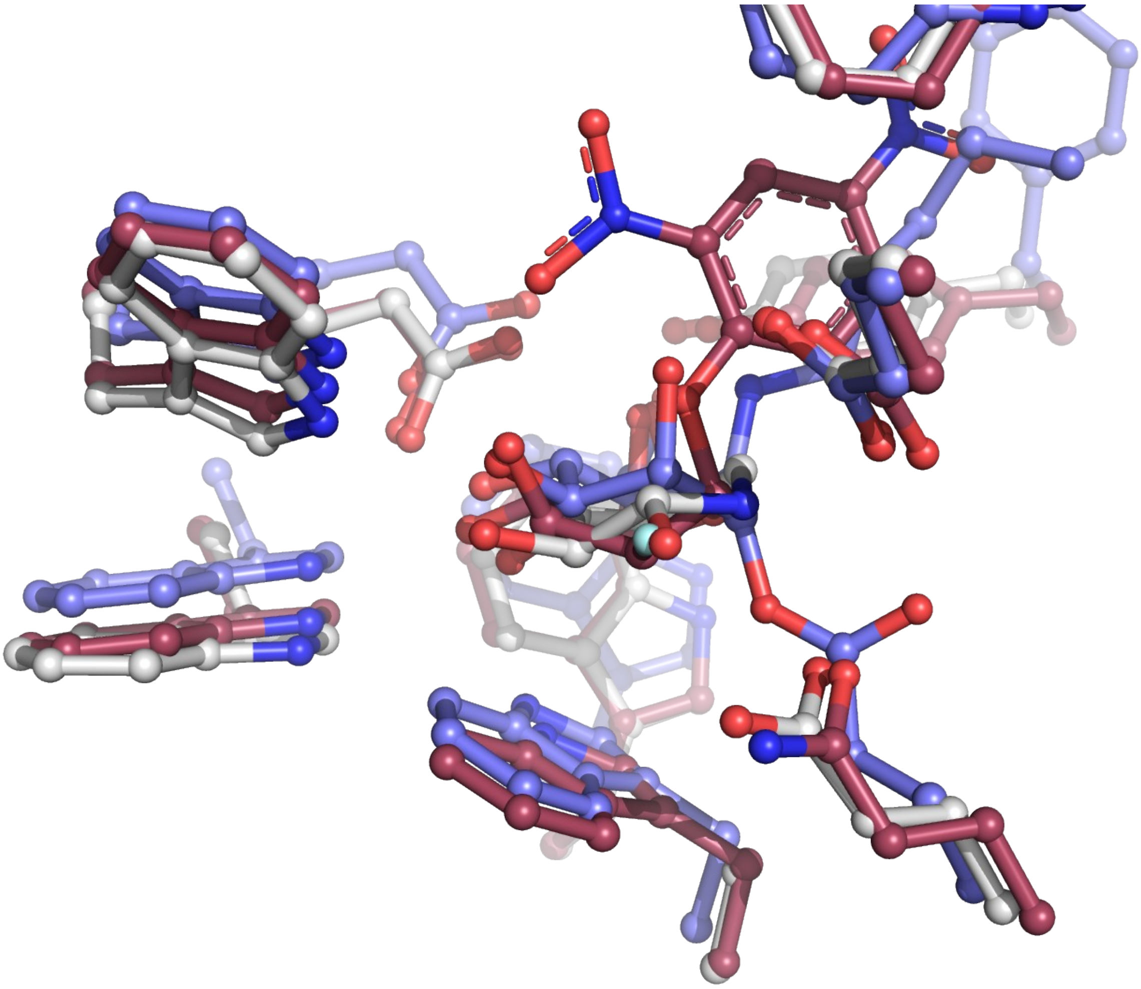

Supplemental figure 3. Superimposition of the structures of *Bt*Man2A labelled with **3** (blue) or bound to noeuromycin (white), or 2FMan (maroon).

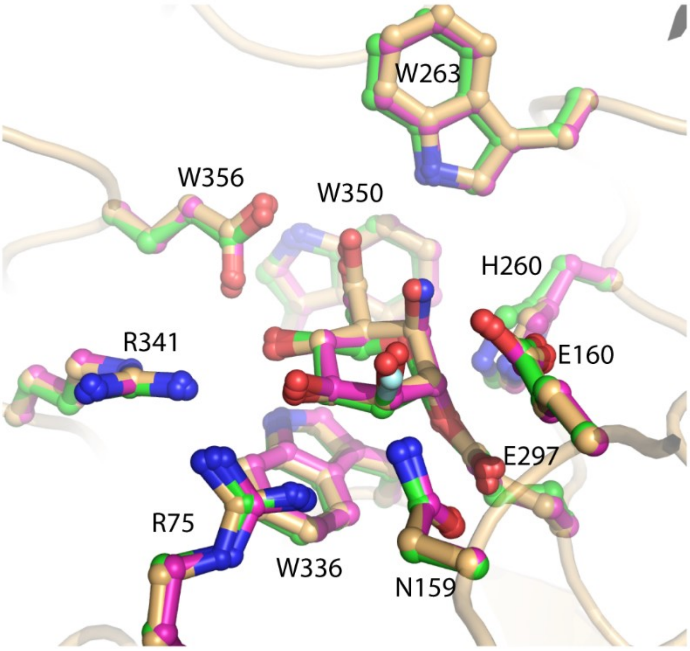

Supplemental figure 4. Superimposition of the structures of *Bs*164 labelled with **1** (light orange), **2** (fuschia), or 2FMan (green). Chain A is shown for all structures.

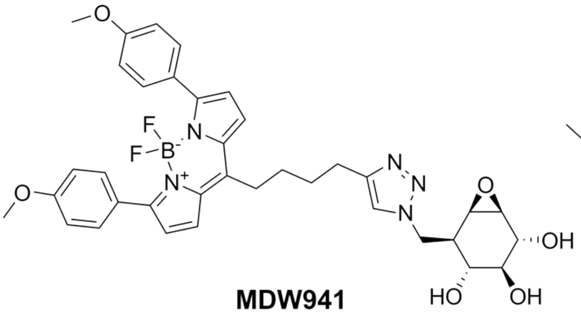

MDW941

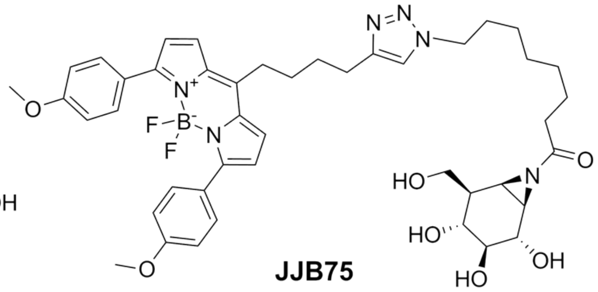

JJB75

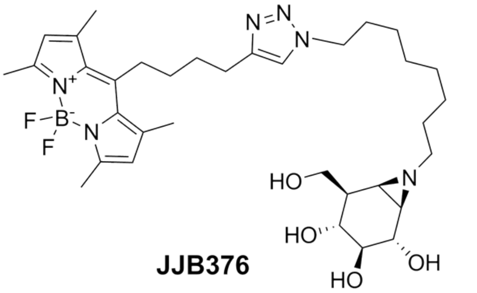

JJB376

Supplemental figure 5. Other ABPs used in this study

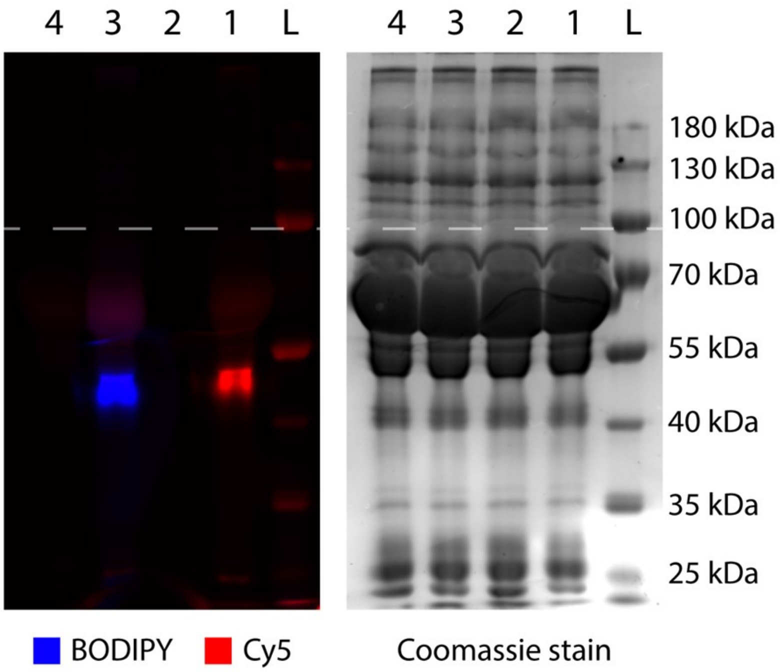

Supplemental figure 6. ABPP of human plasma using probe **4**. Sample 1: plasma treated with probe **4** for 2 hours at 37°C. Sample 2: plasma without probe treatment. Sample 3: plasma stained with 4 following treatment with β-glucosidase probe (**JJB376**) for 1 hour. Sample 4: SDS-treated plasma treated with **4**. L = Pageruler ladder (Thermo). A transparent dashed line indicates the expected migration position of MANBA.

Supplemental table 1. Residual activities (as % of vehicle ctrl) of *Bt*Man2A, *Cm*Man5A, and *Bs*164 following 25 hour treatment with 1 mM of compounds **1**, **2**, or **3** at pH 5.5 (*Bt*Man2A, *Bs*164) or 7.5 (*Cm*Man5A).

Enzyme	Ctrl	Compound 1	Compound 2	Compound 3
***Bt*Man2A**	100	96	98	13
***Cm*Man5A**	100	80	81	15
***Bs*164**	100	24	70	101

Supplemental table 2. Data collection and refinement statistics (molecular replacement)

	*Bt*Man2A	*Bt*Man2A	*Cm*Man5A	*Bs*164	*Bs*164
	Soaked with **2** (PDB 7OP6)	Soaked with **3** (PDB 7OP7)	Labelled with **3** (PDB 7ODJ)	Soaked with **1** (PDB 7OMI)	Soaked with **2** (PDB 7OMS)
**Data collection**					
Space group	$P2_1$	$P2_1$	$P2_12_12$	P1	P1
a, *b*, *c* (Å)	91.9, 116.9, 100.8	90.8, 114.7, 98.6	91.8, 102.2, 50.7	69.5, 104.4 170.8	69.3, 104.0, 170.1
α, β, γ (°)	90.0, 114.1, 90.0	90.0, 112.9, 90.0	90, 90, 90	92.3, 97.4, 106.6	92.3, 97.7, 106.4
Resolution (Å)	92.02-2.05 (2.10-2.05)	67.57-1.85 (1.90-1.85)	68.3-1.30 (1.32-1.30)	65.87-1.76 (1.79-1.76)	99.60-2.05 (2.09-2.05)
R_{meas}	0.102 (1.067)	0.114 (2.258)	0.120 (1.024)	0.092 (2.388)	0.178 (0.950)
R_{pim}	0.049 (0.522)	0.053 (1.004)	0.025 (0.450)	0.066 (1.688)	0.092 (0.507)
$I / \sigma I$	8.9 (1.3)	8.3 (0.7)	21.3 (1.4)	7.6 (0.5)	3.2 (0.9)
Completeness (%)	98.6 (97.9)	99.9 (99.8)	98.8 (84.9)	97.2 (95.8)	98.1 (97.0)
Redundancy	4.1 (4.0)	5.5 (6.0)	21.5 (4.8)	3.1 (3.3)	3.5 (3.5)
$CC_{1/2}$	0.997 (0.535)	0.997 (0.500)	0.999 (0.631)	0.994 (0.302)	0.980 (0.655)
**Refinement**					
Unique reflections	119904 (5938)	158260 (7873)	116329 (4839)	438213 (22062)	276413 (13624)
R_{work} / R_{free}	0.185/0.233	0.189/0.244	0.125/0.158	0.197/0.224	0.223/0.258
No. atoms					
Protein	13688	13943	3463	31565	31275
Ligand/ion	191	181	44	308/6	128/6
Water	904	828	425	2132	795
B-factors					
Protein	36.4	34.7	16.2	37.57	50.13
Ligand/ion	40.1	39.0	24.0	42.5/30.3	54.94/35.03
Water	37.7	34.5	27.2	39.5	43.93
R.m.s. deviations					
Bond lengths (Å)	0.0078	0.0142	0.015	0.008	0.008
Bond angles (°)	1.577	1.881	1.92	1.42	1.44
Ramachandran plot					
Favoured (%)	97.0	96.5	97.0	97.0	96.8
Allowed (%)	99.6	99.5	100	100	100

*Values in parentheses are for highest-resolution shell.

Supplemental References

1 Z. Armstrong and G. J. Davies, Structure and function of Bs164 β-mannosidase from Bacteroides salyersiae the founding member of glycoside hydrolase family GH164, *J. Biol. Chem.*, 2020, **295**, 4316–4326.

2 F. M. V. Dias, F. Vincent, G. Pell, J. A. M. Prates, M. S. J. Centeno, L. E. Tailford, L. M. A. Ferreira, C. M. G. A. Fontes, G. J. Davies and H. J. Gilbert, Insights into the molecular determinants of substrate specificity in glycoside hydrolase family 5 revealed by the crystal structure and kinetics of Cellvibrio mixtus mannosidase 5A, *J. Biol. Chem.*, 2004, **279**, 25517–25526.

3 F. W. Studier, Protein production by auto-induction in high density shaking cultures., *Protein Expr. Purif.*, 2005, **41**, 207–34.

4 L. E. Tailford, V. A. Money, N. L. Smith, C. Dumon, G. J. Davies and H. J. Gilbert, Mannose foraging by Bacteroides thetaiotaomicron: Structure and specificity of the β-mannosidase, BtMan2A, *J. Biol. Chem.*, 2007, **282**, 11291–11299.

5 N. G. S. McGregor, M. Artola, A. Nin-Hill, D. Linzel, M. Haon, J. Reijngoud, A. Ram, M. N. Rosso, G. A. Van Der Marel, J. D. C. Codeé, G. P. Van Wezel, J. G. Berrin, C. Rovira, H. S. Overkleeft and G. J. Davies, Rational Design of Mechanism-Based Inhibitors and Activity-Based Probes for the Identification of Retaining α-l-Arabinofuranosidases, *J. Am. Chem. Soc.*, 2020, **142**, 4648–4662.

6 G. Winter, C. M. C. Lobley and S. M. Prince, Decision making in xia2, *Acta Crystallogr. Sect. D Biol. Crystallogr.*, 2013, **69**, 1260–1273.

7 G. Winter, D. G. Waterman, J. M. Parkhurst, A. S. Brewster, R. J. Gildea, M. Gerstel, L. Fuentes-Montero, M. Vollmar, T. Michels-Clark, I. D. Young, N. K. Sauter and G. Evans, DIALS: Implementation and evaluation of a new integration package, *Acta Crystallogr. Sect. D Struct. Biol.*, 2018, **74**, 85–97.

8 W. Kabsch, XDS, *Acta Crystallogr. Sect. D Biol. Crystallogr.*, 2010, **66**, 125–132.

9 A. J. McCoy, R. W. Grosse-Kunstleve, P. D. Adams, M. D. Winn, L. C. Storoni and R. J. Read, Phaser crystallographic software, *J. Appl. Crystallogr.*, 2007, **40**, 658–674.

10 A. Vagin and A. Teplyakov, Molecular replacement with MOLREP, *Acta Crystallogr. Sect. D Biol. Crystallogr.*, 2010, **66**, 22–25.

11 F. Long, R. A. Nicholls, P. Emsley, S. Gražulis, A. Merkys, A. Vaitkus and G. N. Murshudov, AceDRG: A stereochemical description generator for ligands, *Acta Crystallogr. Sect. D Struct. Biol.*, 2017, **73**, 112–122.

12 A. A. Lebedev, P. Young, M. N. Isupov, O. V. Moroz, A. A. Vagin and G. N. Murshudov, JLigand: A graphical tool for the CCP4 template-restraint library, *Acta Crystallogr. Sect. D Biol. Crystallogr.*, 2012, **68**, 431–440.

13 P. Emsley, B. Lohkamp, W. G. Scott and K. Cowtan, Features and development of Coot, *Acta Crystallogr. Sect. D Biol. Crystallogr.*, 2010, **66**, 486–501.

14 G. N. Murshudov, P. Skubák, A. A. Lebedev, N. S. Pannu, R. A. Steiner, R. A. Nicholls, M. D. Winn, F. Long and A. A. Vagin, REFMAC5 for the refinement of macromolecular crystal structures, *Acta Crystallogr. Sect. D Biol. Crystallogr.*, 2011, **67**, 355–367.

15 C. Ballard, R. Keegan, E. Krissinel, A. Lebedev, V. Uski, D. Waterman and M. Wojdyr, CCP4: a resource for macromolecular crystallography, *Acta Crystallogr. Sect. A Found. Adv.*, 2014, **70**, C1723–C1723.

16 V. B. Chen, W. B. Arendall, J. J. Headd, D. A. Keedy, R. M. Immormino, G. J. Kapral, L. W. Murray, J. S. Richardson and D. C. Richardson, MolProbity: All-atom structure validation for macromolecular crystallography, *Acta Crystallogr. Sect. D Biol. Crystallogr.*, , DOI:10.1107/S0907444909042073.

17 N. Li, C. L. Kuo, G. Paniagua, H. Van Den Elst, M. Verdoes, L. I. Willems, W. A. Van Der Linden, M. Ruben, E. Van Genderen, J. Gubbens, G. P. Van Wezel, H. S. Overkleeft and B. I. Florea, Relative quantification of proteasome activity by activity-based protein profiling and LC-MS/MS, *Nat. Protoc.*, 2013, **8**, 1155–1168.

18 C. Kuo, W. W. Kallemeijn, L. T. Lelieveld, M. Mirzaian, I. Zoutendijk, A. Vardi, A. H. Futerman, A. H. Meijer, H. P. Spaink, H. S. Overkleeft, J. M. F. G. Aerts and M. Artola, *In vivo* inactivation of glycosidases

by conduritol B epoxide and cyclophellitol as revealed by activity-based protein profiling, *FEBS J.*, 2019, **286**, 584–600.

19 D. Lahav, B. Liu, R. J. B. H. N. Van Den Berg, A. M. C. H. Van Den Nieuwendijk, T. Wennekes, A. T. Ghisaidoobe, I. Breen, M. J. Ferraz, C. L. Kuo, L. Wu, P. P. Geurink, H. Ovaa, G. A. Van Der Marel, M. Van Der Stelt, R. G. Boot, G. J. Davies, J. M. F. G. Aerts and H. S. Overkleeft, A Fluorescence Polarization Activity-Based Protein Profiling Assay in the Discovery of Potent, Selective Inhibitors for Human Nonlysosomal Glucosylceramidase, *J. Am. Chem. Soc.*, 2017, **139**, 14192–14197.

20 Z. Armstrong, C. L. Kuo, D. Lahav, B. Liu, R. Johnson, T. J. M. Beenakker, C. De Boer, C. S. Wong, E. R. Van Rijssel, M. F. Debets, B. I. Florea, C. Hissink, R. G. Boot, P. P. Geurink, H. Ovaa, M. Van Der Stelt, G. M. Van Der Marel, J. D. C. Codée, J. M. F. G. Aerts, L. Wu, H. S. Overkleeft and G. J. Davies, Manno-epi-cyclophellitols Enable Activity-Based Protein Profiling of Human α-Mannosidases and Discovery of New Golgi Mannosidase II Inhibitors, *J. Am. Chem. Soc.*, 2020, **142**, 13021–13029.

21 S. P. Schröder, J. W. Van De Sande, W. W. Kallemeijn, C. L. Kuo, M. Artola, E. J. Van Rooden, J. Jiang, T. J. M. Beenakker, B. I. Florea, W. A. Offen, G. J. Davies, A. J. Minnaard, J. M. F. G. Aerts, J. D. C. Codée, G. A. Van Der Marel and H. S. Overkleeft, Towards broad spectrum activity-based glycosidase probes: Synthesis and evaluation of deoxygenated cyclophellitol aziridines, *Chem. Commun.*, 2017, **53**, 12528–12531.
